# Clinical, Oncological, and Hematological Determinants of In-Hospital Mortality in Critically Ill Patients with Advanced Neoplasia: A Retrospective Cohort Study

**DOI:** 10.3390/jcm15218669

**Published:** 2026-11-06

**Authors:** Valeriu Plătică, Iulia Chiscop, Adina Plătică, Magdalena Miulescu, Corina-Cecilia Manole Pălivan, Anamaria Hărăbor, Dorel Firescu, Mihaela Lungu, Ingrid-Andrada Vasilache, Laura-Florentina Rebegea

**Affiliations:** 1 Faculty of Medicine and Pharmacy, “Dunărea de Jos” University of Galați, 800008 Galați, Romaniamagdalena.miulescu@ugal.ro (M.M.);; 2 Comrat State University, MD-3805 Comrat, Moldova; 3 Faculty of Medicine and Biological Sciences, ‘Ștefan cel Mare’ University, 720229 Suceava, Romania

**Keywords:** critically ill neoplasia patients, intensive care unit, in-hospital mortality, liver metastases, neutrophil-to-lymphocyte ratio, systemic inflammation, prognostic biomarkers

## Abstract

**Background/Objectives**: Short-term prognostication remains challenging in critically ill patients with neoplasia. We aimed to retrospectively evaluate the clinical, oncological, and hematological factors associated with in-hospital mortality and its timing in 120 patients with neoplasia admitted to intensive care unit (ICU). **Methods**: This retrospective cohort included 120 patients admitted to an intensive care unit (ICU). In-hospital mortality was analyzed using Firth penalized logistic regression with adjustment for Acute Physiology and Chronic Health Evaluation II (APACHE II) scores recorded at ICU admission. Time to death was analyzed using Cox regression and Fine–Gray competing-risk regression. Predictive performance was internally validated using 1000 bootstrap resamples, with calibration and exploratory decision-curve analyses. **Results**: Mortality was 80.0%. After APACHE II adjustment, hepatic metastases remained associated with mortality (adjusted odds ratio [aOR] 7.87, 95% confidence interval [CI] 2.41–29.44), whereas platelet count was inversely associated (aOR 0.82 per 50 × 10^9^/L, 95% CI 0.69–0.94). Neutrophil-to-lymphocyte ratio (NLR) was not significantly associated with binary mortality. In APACHE II-adjusted Fine–Gray analysis, NLR (subdistribution hazard ratio [sHR] 1.20 per doubling, 95% CI 1.03–1.40) and platelet count (sHR 0.90, 95% CI 0.84–0.97) were associated with the cumulative incidence of death. Adding platelet count increased the optimism-corrected area under the receiver operating characteristic curve (AUROC) from 0.743 to 0.799 in the original clinical model (ΔAUROC 0.056, 95% CI 0.002–0.146); after APACHE II adjustment, the gain was uncertain (0.745 to 0.793; ΔAUROC 0.048, 95% CI −0.0003–0.128). **Conclusions**: Hepatic metastases and platelet count retained prognostic associations after severity adjustment.

## 1. Introduction

Critically ill patients with neoplasia represent an increasingly important population in contemporary intensive care, reflecting both advances in oncologic treatment and a broader shift away from considering malignancy itself a contraindication to intensive care unit (ICU) admission [1]. The introduction of targeted therapies, immunotherapy, and improved supportive strategies has expanded the population of patients who may benefit from intensive care, including those admitted with potentially reversible acute complications or treatment-related toxicities [2]. Nevertheless, short-term outcomes remain highly heterogeneous. Contemporary cohorts continue to report substantial ICU and in-hospital mortality, particularly among patients requiring unplanned admission or presenting with advanced malignancy and acute organ dysfunction [3,4]. Accurate early prognostication therefore remains central to clinical decision-making, communication with patients and families, and appropriate allocation of intensive care resources.

Prognosis in this setting cannot be inferred from the diagnosis of neoplasia alone. Increasing evidence indicates that outcomes reflect the interaction between acute physiological derangement, pre-existing functional status, and the underlying oncologic disease burden [4,5]. Requirements for invasive mechanical ventilation, vasopressor support, renal replacement therapy, and the presence of severe organ dysfunction have repeatedly been associated with adverse short-term outcomes, whereas performance status and cancer progression contribute additional prognostic information [1,3].

In a large cohort of patients with solid malignancies requiring unplanned ICU admission, Vigneron et al. identified metastatic disease and cancer progression among the independent determinants of ICU mortality [1]. Similarly, analyses by Marchini Dias da Silva et al. [6] and Van der Zee et al. [7] emphasized the prognostic importance of functional status, illustrating that the oncologic context remains relevant even when acute critical illness is taken into account.

Within this framework, metastatic disease is commonly treated as a binary characteristic, potentially obscuring prognostic heterogeneity related to the anatomical distribution of metastatic burden. Metastatic disease has been independently associated with ICU mortality across several populations of critically ill patients with cancer [1,8]. However, substantially less is known about the prognostic relevance of individual metastatic sites. García de Herreros et al. reported an association between liver metastases and in-hospital mortality in a cohort of patients with solid tumors admitted to the ICU, although hepatic involvement was not retained as an independent determinant in their adjusted analysis [9].

Systemic inflammation represents another potentially informative dimension of prognosis in critically ill patients with cancer. Cancer-associated inflammation, acute physiological stress, infection, and treatment-related effects may converge to produce neutrophilia, lymphopenia, platelet activation, and alterations in monocyte-mediated immune responses [10,11]. These readily measurable changes form the basis of several inexpensive composite indices, including the neutrophil-to-lymphocyte ratio (NLR), platelet-to-lymphocyte ratio (PLR), lymphocyte-to-monocyte ratio (LMR), systemic immune-inflammation index (SII), and prognostic nutritional index (PNI). Their prognostic relevance has been investigated across general oncology populations. Meta-analyses and large cohort studies have associated elevated NLR, PLR, and SII, as well as lower LMR, with adverse oncologic outcomes across multiple tumor types and treatment settings [12,13].

Whether these associations translate into clinically useful prognostic information in the ICU is considerably less certain. Tunay et al., in patients with lung cancer admitted to intensive care, found that NLR, PLR, LMR, and SII did not retain independent associations with mortality after multivariable adjustment, despite signals for some indices in unadjusted analyses [14]. Similar inconsistencies have been reported in other critically ill oncologic populations [15,16].

Moreover, demonstrating an association with outcome does not necessarily establish incremental prognostic value. Few studies have specifically examined whether inflammation-based indices meaningfully improve discrimination beyond readily available clinical and oncologic characteristics. A biomarker may provide limited discrimination between survivors and non-survivors while still identifying patients who experience an earlier fatal course. This question has received comparatively little attention in ICU oncology, where high and early event rates may make the distinction particularly important [3].

The present study aimed to investigate clinical, oncological, and biological factors associated with in-hospital mortality and time to death in critically ill patients with cancer. We examined the prognostic relevance of hepatic metastatic involvement, the reason for ICU admission, and routinely available hematological markers, including NLR, PLR, SII, and platelet count, and assessed whether these markers improved model performance. Competing-risk analyses and changes in renal, coagulation, and hepatic parameters during hospitalization were also examined.

## 2. Materials and Methods

### 2.1. Study Design and Population

This retrospective observational cohort study included 120 adult patients with active malignancy admitted to the intensive care unit (ICU) of Saint Apostle Andrew County Clinical Emergency Hospital, Galati, Romania between January 2020 to May 2024. The primary outcome was in-hospital death. In addition to binary mortality, time from ICU admission to in-hospital death was analyzed to investigate factors associated with the timing of death.

Patients were eligible if they were aged ≥18 years, had an active advanced malignancy, and required ICU admission during the study period. Patients younger than 18 years, those without a confirmed malignancy, patients whose malignancy was in remission, and those with insufficient clinical or laboratory data for the planned analyses were excluded.

Clinical, oncological, laboratory, APACHE II (Acute Physiology and Chronic Health Evaluation II) scores at admission in the ICU and treatment-related information were extracted from the available medical records. Demographic and oncological variables included age, sex, primary tumor site, presence of metastatic disease, hepatic metastatic involvement, and cachexia. Active malignancy was defined as the presence of documented cancer that was not in remission at the time of ICU admission. Advanced neoplasia was defined as locally advanced or metastatic malignant disease. Hepatic metastatic involvement was defined as documented liver metastases present before or at ICU admission, based on available oncological and imaging records.

Acute clinical presentation was characterized according to the primary reason for ICU admission, which was recorded as hemorrhage, neurological deterioration, postoperative admission, respiratory failure, septic shock, or other shock. For multivariable analyses, the six original admission categories were consolidated into three groups: neurological deterioration, respiratory failure, and other critical illness. The latter included hemorrhage, septic shock, other shock, and postoperative admission. Although these conditions were clinically heterogeneous, aggregation was necessary to limit model complexity and avoid unstable parameter estimates arising from small category-specific sample sizes and the limited number of survivors.

For the primary multivariable analyses, these categories were grouped into neurological deterioration, respiratory failure, and other critical illness, the latter comprising hemorrhage, septic shock, other shock, and postoperative admission, to preserve model parsimony.

Variables available at ICU admission were considered baseline variables and were analyzed separately from measurements and interventions occurring subsequently during the ICU stay. Organ-support and treatment variables included vasopressor requirement, invasive mechanical ventilation, non-invasive ventilation, antibiotic therapy, and corticosteroid therapy. The available treatment variables were used to characterize the clinical course but were not included as covariates in the baseline prognostic models.

The distinction between baseline characteristics and post-admission variables was maintained throughout the analyses to avoid incorporating subsequent clinical deterioration or treatment requirements as baseline prognostic information.

### 2.2. Laboratory Variables and Inflammation-Based Indices

Baseline hematological and biochemical variables included hemoglobin (HGB), white blood cell count (WBC), platelet count (PLT), serum creatinine, and urea. Analyses were based on verified laboratory measurements obtained at ICU admission. Missing observations were retained as missing and were not replaced by zero.

Inflammation-based indices were recalculated from the verified baseline peripheral blood cell counts. The neutrophil-to-lymphocyte ratio (NLR) was calculated as the absolute neutrophil count divided by the absolute lymphocyte count, the platelet-to-lymphocyte ratio (PLR) as the platelet count divided by the absolute lymphocyte count, and the systemic immune-inflammation index (SII) as platelet count × absolute neutrophil count/absolute lymphocyte count. NLR, PLR, and SII were calculable for all 120 patients.

Because of their right-skewed distributions and to provide clinically interpretable effect estimates, NLR, PLR, and SII were log_2_-transformed for regression analyses. Accordingly, their odds ratios (ORs) and hazard ratios (HRs) represent the change in outcome associated with a doubling of the corresponding index. Platelet count was modeled per 50 × 10^9^/L increase, and WBC was modeled per 5 × 10^9^/L increase in time-to-event analyses.

### 2.3. Assessment of Post-Admission Biological Evolution

Post-admission biological evolution was evaluated separately from baseline prognostic factors. For serum creatinine, urea, international normalized ratio (INR), and aspartate aminotransferase (AST), the verified baseline measurement and the final valid measurement were identified. Final values were defined as the last available valid measurement recorded during the ICU/hospital course before death or discharge.

Absolute within-patient change was calculated as the final post-admission value minus the corresponding baseline value. Baseline, final, and change values were compared between survivors and non-survivors. These longitudinal variables were used to characterize the biological evolution of critical illness and were not incorporated into the baseline prognostic models.

### 2.4. Statistical Analysis

Continuous variables were summarized as median and interquartile range (IQR), given their distributions, and categorical variables as counts and percentages. Baseline characteristics were compared between survivors and non-survivors using the Wilcoxon rank-sum test for continuous variables and the χ^2^ test or Fisher exact test for categorical variables, as appropriate. Statistical tests were two-sided, and *p* < 0.05 was considered statistically significant.

The primary regression endpoint was in-hospital mortality. Given the marked outcome imbalance, with 96 deaths and 24 survivors, associations with in-hospital death were estimated using Firth penalized-likelihood logistic regression. Univariable Firth models were fitted for baseline demographic, oncological, acute clinical presentation, laboratory, and inflammation-based variables. Primary reason for ICU admission was evaluated using the three-category ICU admission phenotype (neurological deterioration, respiratory failure, and other critical illness), with neurological deterioration as the reference category. Age was scaled per 10-year increase and platelet count per 50 × 10^9^/L increase. NLR, PLR, and SII were entered on the log_2_ scale and interpreted per doubling. Odds ratios (ORs) with 95% confidence intervals (CIs) were reported.

The principal multivariable analysis used a core adjustment model comprising age, hepatic metastatic involvement, and the primary reason for ICU admission, in order to account for both oncological burden and acute clinical presentation while limiting model complexity relative to the sample size and number of survivors. NLR, PLR, and SII were subsequently added individually to the core model. An additional model evaluated platelet count using the same adjustment set. A combined model incorporating both NLR and platelet count was examined as an exploratory analysis. Adjusted odds ratios (aORs) with 95% confidence intervals (CIs) were reported.

Time to in-hospital death was analyzed using Cox proportional-hazards regression, with patients discharged alive censored at hospital discharge. Univariable Cox models were fitted for the same baseline demographic, oncological, acute clinical presentation, laboratory, and inflammation-based variables evaluated in the binary mortality analysis. Primary reason for ICU admission was represented by the same three-category ICU admission phenotype, with neurological deterioration as the reference category. Age, platelet count, and the inflammation-based indices were scaled or transformed as described above. Hazard ratios (HRs) with 95% CIs were reported.

The multivariable Cox analyses used the same core adjustment model comprising age, hepatic metastatic involvement, and primary reason for ICU admission. The primary reason for ICU admission was categorized as neurological deterioration, respiratory failure, or other critical illness, with neurological deterioration as the reference category. NLR, PLR, SII, and platelet count were subsequently evaluated individually in extended models. A combined model incorporating both NLR and platelet count was examined as an exploratory analysis. Hazard ratios (HRs) with 95% confidence intervals (CIs) were reported. The proportional-hazards assumption was evaluated using Schoenfeld residuals and global proportional-hazards tests.

To assess whether the observed associations were disproportionately influenced by patients admitted in an immediately preterminal state, sensitivity analyses were performed after sequentially excluding patients who died within 24 and 48 h of ICU admission. The combined Firth model including age, hepatic metastatic involvement, ICU admission phenotype, NLR, and platelet count was refitted in each restricted cohort. These analyses included 114 patients (90 deaths) after exclusion of deaths within 24 h and 103 patients (79 deaths) after exclusion of deaths within 48 h, compared with 120 patients (96 deaths) in the full cohort. Effect estimates were compared across analyses to assess the consistency of the observed associations.

Internal validation of the Firth logistic regression models was performed using 1000 bootstrap resamples. Model performance was assessed using the area under the receiver-operating-characteristic curve (AUROC) for discrimination, the Brier score for overall prediction error, and the calibration slope. Bootstrap optimism was estimated as the difference between performance in each bootstrap sample and performance of the corresponding bootstrap-fitted model when applied to the original cohort. Optimism-corrected estimates were obtained by subtracting the mean optimism from the apparent performance.

Baseline values, final post-admission values, and absolute within-patient changes were compared between survivors and non-survivors using the Wilcoxon rank-sum test. Within-patient baseline-to-final changes were additionally evaluated using the paired Wilcoxon signed-rank test. To account for multiple testing of longitudinal change, the Benjamini–Hochberg procedure was applied across the four between-group comparisons of absolute change in creatinine, urea, INR, and AST. These longitudinal variables were analyzed as markers of in-hospital biological evolution and were not included as predictors in the baseline prognostic models.

Cumulative incidence functions were estimated treating discharge alive as a competing event. Fine–Gray regression was used to estimate subdistribution hazard ratios (sHRs) with 95% CIs, which quantify the association between baseline predictors and the cumulative incidence of in-hospital death while retaining patients discharged alive within the competing-risk framework. This analysis complemented the cause-specific Cox models, in which patients discharged alive were censored. The Fine–Gray subdistribution hazard regression was performed using the combined model including age, hepatic metastatic involvement, ICU admission phenotype, NLR, and platelet count. Neurological deterioration was used as the reference category for ICU admission phenotype.

To further account for potential confounding by acute physiological severity, additional multivariable analyses incorporating APACHE II were performed. APACHE II was modeled as a continuous variable, scaled per five-point increase.

Severity-adjusted Firth penalized logistic regression models were constructed using APACHE II, hepatic metastatic involvement, and primary reason for ICU admission, categorized as neurological deterioration, respiratory failure, or other critical illness, with neurological deterioration serving as the reference category. Platelet count and NLR were subsequently incorporated individually and jointly to evaluate their associations with in-hospital mortality beyond standardized physiological severity.

Because age is already incorporated into APACHE II, it was not included separately in the principal severity-adjusted models. Additional sensitivity analyses including age were performed to evaluate the stability of the observed associations.

The time-to-event analyses were also repeated after adjustment for APACHE II. Cause-specific Cox proportional-hazards regression models incorporated APACHE II, hepatic metastatic involvement, and ICU admission phenotype, with NLR, PLR, SII, and platelet count evaluated individually in extended models.

A combined model including both NLR and platelet count was additionally fitted. Patients discharged alive were censored at hospital discharge. The proportional-hazards assumption was assessed using Schoenfeld residuals and global proportional-hazards tests.

To account for discharge alive as a competing event, additional Fine–Gray subdistribution hazard regression analyses were performed incorporating APACHE II, hepatic metastatic involvement, ICU admission phenotype, platelet count, and NLR. In-hospital death was defined as the event of interest, whereas discharge alive was treated as the competing event. A severity-adjusted clinical model and an extended model incorporating platelet count were also evaluated. Additional sensitivity analyses included age as a separate covariate. The sHRs with 95% CI were reported.

The robustness of the severity-adjusted associations was further evaluated through sensitivity analyses excluding patients who died within 24 and 48 h of ICU admission. The combined Firth penalized logistic regression model incorporating APACHE II, hepatic metastatic involvement, ICU admission phenotype, platelet count, and NLR was refitted in the restricted cohorts, retaining patients who survived hospitalization. Complementary landmark analyses were performed for Cox and Fine–Gray regression at 24 and 48 h after ICU admission. These analyses were restricted to patients who remained alive and hospitalized beyond the respective landmark, with subsequent follow-up time calculated from the landmark. The same severity-adjusted covariate structure was retained to examine the stability of the prognostic associations across the restricted populations.

The incremental predictive contribution of hematological markers was assessed by comparing the original clinical Core model and the APACHE II-adjusted Core model with extended specifications incorporating platelet count, NLR, or both markers. PLR and SII were additionally evaluated in separate models based on the original clinical adjustment set.

Internal validation was performed using 1000 paired bootstrap resamples, with identical resampling indices applied across candidate models. Model discrimination was assessed using the AUROC, and overall prediction error was evaluated using the Brier score. Incremental predictive performance was quantified through paired differences in optimism-corrected AUROC and Brier scores. Approximate 95% CI for individual performance estimates and pairwise differences were obtained.

Model calibration was assessed using calibration-in-the-large, logistic recalibration intercepts, and calibration slopes. Calibration-in-the-large was estimated by fitting a logistic regression model with the predicted log-odds included as an offset, whereas recalibration intercepts and slopes were obtained by regressing observed outcomes on predicted log-odds. Optimism-corrected calibration estimates were calculated using the bootstrap procedure described above.

Calibration was additionally examined graphically by comparing predicted probabilities with observed mortality across five approximately equally sized groups of predicted risk, with binomial 95% CIs for the observed proportions.

Potential clinical utility was explored using decision-curve analysis across threshold probabilities ranging from 0.50 to 0.85, in increments of 0.025. Model-based high-risk classification was compared with strategies classifying all patients or no patients as high risk of in-hospital death. Net benefit was estimated at each threshold and corrected for optimism using paired bootstrap resampling. Incremental net benefit was calculated as the difference between each extended model and its corresponding Core model, with approximate 95% CIs obtained.

Statistical analyses were performed using R (version 4.6.1, R Foundation for Statistical Computing, Vienna, Austria).

### 2.5. Ethical Considerations

The study was conducted in accordance with the Declaration of Helsinki. The protocol was approved by the local institutional ethics committee of Saint Apostle Andrew County Clinical Emergency Hospital, Galati, Romania (14019/20 June 2024), and written informed consent was waived from all participants due to the retrospective study design.

## 3. Results

The study included 120 critically ill patients with malignancy, of whom 96 (80.0%) died during hospitalization and 24 (20.0%) survived (Table 1). The median age was 66 years (IQR 59–73), with no significant difference between survivors and non-survivors (63 vs. 67 years, respectively; *p* = 0.174). Women represented 36.1% of patients, with a similar distribution between outcome groups (33.3% vs. 36.8%; *p* = 0.816).

Colorectal (22.5%) and pulmonary (20.8%) malignancies were the most frequent primary tumor sites, and the overall distribution of tumor localization did not differ significantly according to survival status (*p* = 0.463). Metastatic disease was present in 115 patients (95.8%) and was more frequent among non-survivors than survivors (97.9% vs. 87.5%), although the difference did not reach conventional statistical significance (*p* = 0.054). On the other hand, hepatic metastatic involvement showed a marked association with outcome, being present in 85.4% of non-survivors compared with 54.2% of survivors (*p* = 0.002). Cachexia was also more frequent among patients who died during hospitalization (59.4% vs. 33.3%; *p* = 0.038).

Baseline hematological and biochemical parameters were available for all 120 patients following verification of the admission laboratory measurements. Median hemoglobin was 10.1 g/dL (IQR 8.10–12.2), median WBC count was 11.5 × 10^9^/L (IQR 6.74–18.2), and median platelet count was 226 × 10^9^/L (IQR 147–365). Baseline hemoglobin, WBC, creatinine, and urea did not differ significantly between survivors and non-survivors (all *p* > 0.05). Platelet count was numerically lower among non-survivors than survivors (218 [136–352] vs. 274 [213–424] × 10^9^/L), although the unadjusted difference did not reach conventional statistical significance (*p* = 0.066).

The recalculated inflammation-based indices were available for all 120 patients. Median NLR was 8.97 (IQR 5.34–15.4), median PLR was 217 (IQR 118–384), and median SII was 2159 (IQR 827–4376). NLR was numerically higher among non-survivors than survivors (9.17 [5.83–17.1] vs. 8.13 [3.15–12.6]), but the difference was not statistically significant (*p* = 0.165). PLR and SII were likewise comparable between outcome groups (*p* = 0.966 and *p* = 0.750, respectively).

Organ-support requirements during the ICU stay differed markedly according to outcome. Vasopressors were required in 90/96 (93.8%) non-survivors compared with 5/24 (20.8%) survivors (*p* < 0.001), while invasive mechanical ventilation was required in 68/96 (70.8%) and 2/24 (8.3%), respectively (*p* < 0.001). NIV, antibiotic use, and corticosteroid use did not differ significantly between groups. APACHE II scores were available for all 120 patients, with a median of 20.0 (IQR 14.75–24.25) and did not significantly differ between groups (*p* = 0.347).

Neurological deterioration was recorded in 22 patients (10 survivors and 12 non-survivors), respiratory failure in 67 (10 survivors and 57 non-survivors), hemorrhage in 11 (2 survivors and 9 non-survivors), septic shock in 16 (1 survivor and 15 non-survivors), other shock in 3 (all non-survivors), and postoperative admission in 1 patient (survivor).

In univariable Firth penalized logistic regression, hepatic metastatic involvement showed the strongest association with in-hospital mortality, with patients presenting liver metastases having approximately five-fold higher odds of death than those without hepatic metastatic involvement (OR 4.85, 95% CI 1.85–12.89; *p* = 0.002) (Table 2). Age was not significantly associated with mortality when modeled per 10-year increase (OR 1.35, 95% CI 0.89–2.06; *p* = 0.155).

Among baseline hematological parameters, higher platelet count was associated with lower odds of in-hospital mortality. Each 50 × 10^9^/L increase in platelet count was associated with 12% lower odds of death (OR 0.88, 95% CI 0.77–1.00; *p* = 0.045). Hemoglobin and WBC were not significantly associated with mortality (*p* = 0.657 and *p* = 0.385, respectively). Similarly, neither baseline creatinine nor urea showed a significant association with in-hospital death (*p* = 0.644 and *p* = 0.131, respectively).

Among the inflammation-based indices, NLR showed the strongest univariable signal, with each doubling associated with 33% higher odds of in-hospital death; however, the confidence interval included the null and the association did not reach conventional statistical significance (OR 1.33, 95% CI 0.98–1.82; *p* = 0.063). PLR and SII were not significantly associated with binary mortality (OR 1.05, 95% CI 0.77–1.40; *p* = 0.752 and OR 1.07, 95% CI 0.88–1.29; *p* = 0.486, respectively).

Primary reason for ICU admission was also associated with binary in-hospital mortality. Using neurological deterioration as the reference category, admission for respiratory failure was associated with higher odds of death (OR 4.60, 95% CI 1.61–13.50; *p* = 0.004), as was admission for other critical illness (OR 5.13, 95% CI 1.48–20.40; *p* = 0.009).

**Figure 1:**
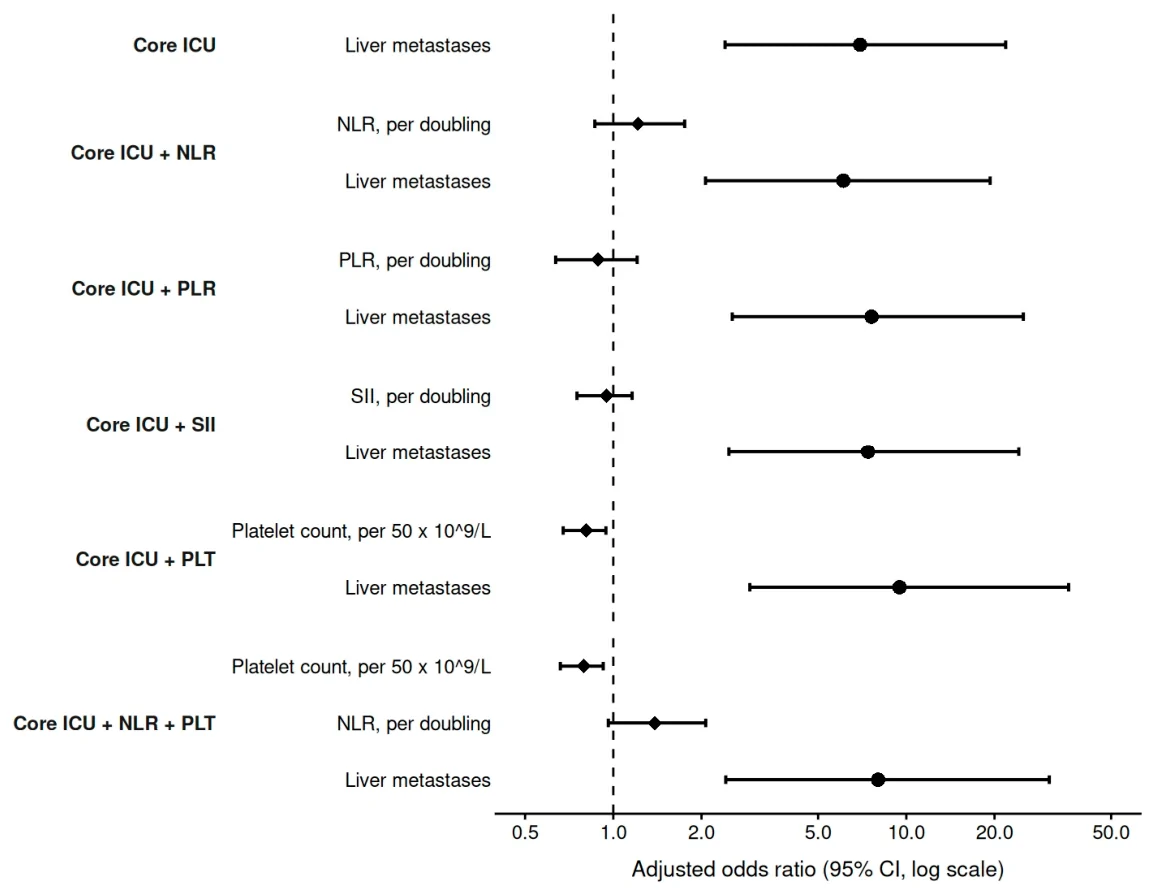
Multivariable Firth logistic regression models for in-hospital mortality.

In multivariable Firth penalized logistic regression, hepatic metastatic involvement remained consistently and independently associated with in-hospital mortality after adjustment for age and the primary reason for ICU admission (Table 3 and Figure 1). In the Core ICU model, liver metastases were associated with substantially higher odds of in-hospital death (aOR 6.96, 95% CI 2.41–21.80; *p* < 0.001). Compared with neurological deterioration, respiratory failure (aOR 6.78, 95% CI 2.10–24.10; *p* = 0.001) and other critical illness (aOR 6.85, 95% CI 1.75–32.10; *p* = 0.005) as the primary reason for ICU admission were also independently associated with mortality, whereas age was not (aOR 1.17 per 10-year increase, 95% CI 0.75–1.83; *p* = 0.491).

The association with hepatic metastatic involvement remained robust after individual addition of the inflammation-based indices. In the Core ICU + NLR model, liver metastases remained independently associated with mortality (aOR 6.09, 95% CI 2.06–19.30; *p* = 0.001), whereas NLR was not independently associated with binary mortality (aOR 1.21 per doubling, 95% CI 0.87–1.75; *p* = 0.264). Neither PLR nor SII was independently associated with in-hospital mortality (aOR 0.89, 95% CI 0.64–1.21; *p* = 0.448 and aOR 0.95, 95% CI 0.75–1.16; *p* = 0.603, respectively). Across these models, hepatic metastatic involvement and ICU admission phenotype remained consistently associated with mortality.

In the Core ICU + PLT model, higher baseline platelet count was independently associated with lower odds of in-hospital mortality. Each 50 × 10^9^/L increase in platelet count was associated with a 19% reduction in the odds of death (aOR 0.81, 95% CI 0.67–0.94; *p* = 0.006). In this model, hepatic metastatic involvement remained strongly associated with mortality (aOR 9.48, 95% CI 2.92–35.80; *p* < 0.001), as did respiratory failure (aOR 10.40, 95% CI 2.89–44.30; *p* < 0.001) and other critical illness (aOR 7.54, 95% CI 1.83–38.10; *p* = 0.005).

In the model that incorporated NLR and platelet count, higher platelet count retained an independent inverse association with mortality (aOR 0.79 per 50 × 10^9^/L increase, 95% CI 0.66–0.92; *p* = 0.002), whereas NLR showed a positive but non-significant association (aOR 1.39 per doubling, 95% CI 0.96–2.07; *p* = 0.080). Hepatic metastatic involvement remained independently associated with mortality (aOR 8.01, 95% CI 2.42–30.80; *p* < 0.001), together with respiratory failure (aOR 10.40, 95% CI 2.82–46.00; *p* < 0.001) and other critical illness (aOR 6.57, 95% CI 1.54–34.40; *p* = 0.010). Age was not independently associated with mortality in the combined model (*p* = 0.146).

To determine whether the associations observed in the original multivariable models persisted after accounting for acute physiological severity, additional Firth penalized logistic regression analyses incorporating APACHE II were performed (Table 4). Hepatic metastatic involvement remained strongly associated with in-hospital mortality (aOR 7.87, 95% CI 2.41–29.44; *p* < 0.001). Compared with neurological deterioration, respiratory failure (aOR 11.29, 95% CI 3.04–50.78; *p* < 0.001) and other critical illness (aOR 7.31, 95% CI 1.75–36.97; *p* = 0.006) were also independently associated with increased mortality odds. Higher baseline platelet count retained an inverse association with mortality (aOR 0.82 per 50 × 10^9^/L increase, 95% CI 0.69–0.94; *p* = 0.005).

On the other hand, neither APACHE II (aOR 1.05 per five-point increase, 95% CI 0.71–1.56; *p* = 0.791) nor NLR (aOR 1.29 per doubling, 95% CI 0.90–1.89; *p* = 0.168) showed statistically significant associations in the combined model. Additional adjustment for age yielded consistent findings for hepatic metastatic involvement (aOR 7.87, 95% CI 2.39–30.11; *p* < 0.001) and platelet count (aOR 0.80, 95% CI 0.66–0.93; *p* = 0.002).

In univariable Cox proportional-hazards analyses, hepatic metastatic involvement, WBC, platelet count, baseline creatinine, urea, and NLR were associated with time to in-hospital death (Table 5). Liver metastases were associated with an 85% higher hazard of death (HR 1.85, 95% CI 1.05–3.27; *p* = 0.035), while each 5 × 10^9^/L increase in WBC was associated with a 13% higher hazard (HR 1.13, 95% CI 1.04–1.23; *p* = 0.004). Higher platelet count showed an inverse association, with each 50 × 10^9^/L increase corresponding to a 7% lower hazard of death (HR 0.93, 95% CI 0.87–0.99; *p* = 0.026). Higher baseline creatinine and urea were also associated with earlier death (HR 1.17, 95% CI 1.02–1.33; *p* = 0.020 and HR 1.01, 95% CI 1.00–1.01; *p* < 0.001, respectively).

Among the inflammation-based indices, NLR was associated with time to death, with each doubling corresponding to a 20% higher hazard of in-hospital death (HR 1.20, 95% CI 1.02–1.41; *p* = 0.028). In contrast, PLR and SII were not significantly associated with the timing of death (*p* = 0.783 and *p* = 0.816, respectively).

The association between primary reason for ICU admission and the timing of death was less uniform. Using neurological deterioration as the reference category, respiratory failure was not associated with time to in-hospital death (HR 1.25, 95% CI 0.67–2.33; *p* = 0.483), whereas other critical illness was associated with a higher hazard of earlier death (HR 2.05, 95% CI 1.04–4.05; *p* = 0.039).

**Figure 2:**
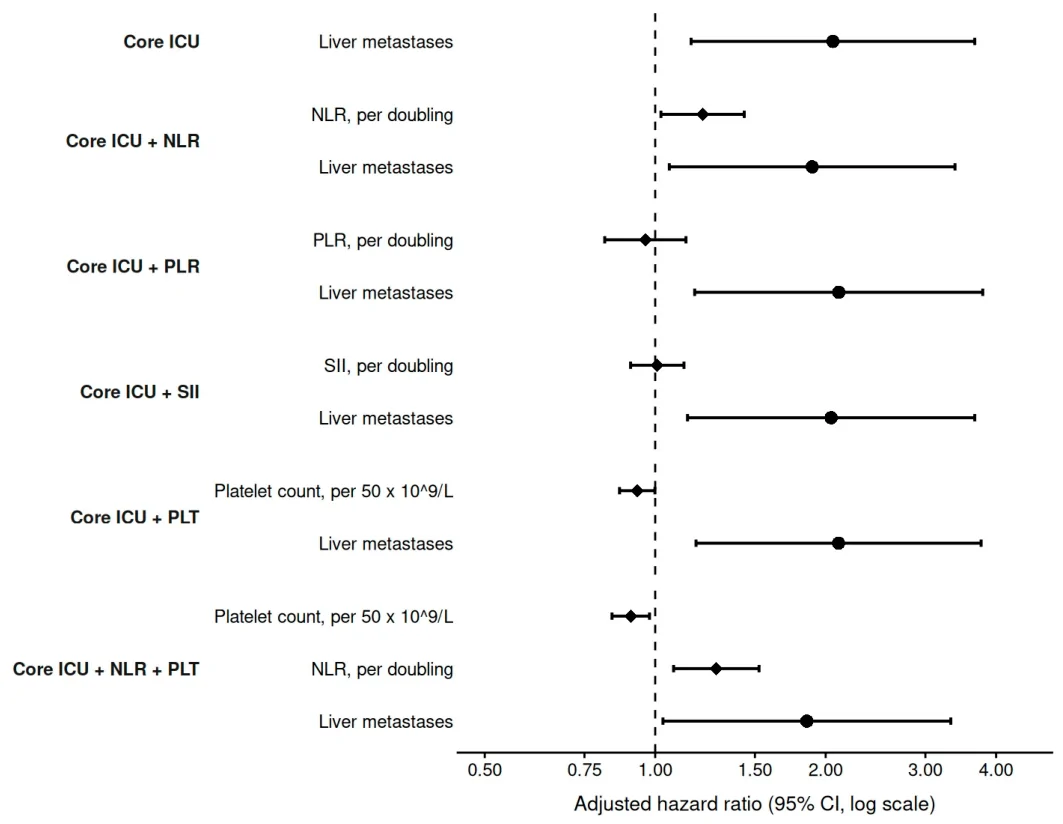
Adjusted associations with time to in-hospital death across multivariable Cox proportional-hazards models.

In the multivariable Cox analysis, hepatic metastatic involvement remained independently associated with earlier in-hospital death after adjustment for age and primary reason for ICU admission (Table 6 and Figure 2). In the Core ICU model, liver metastases were associated with a higher hazard of death (aHR 2.06, 95% CI 1.16–3.67; *p* = 0.014), whereas age was not independently associated with outcome (aHR 1.11 per 10-year increase, 95% CI 0.92–1.35; *p* = 0.289). Compared with neurological deterioration, other critical illness as the primary reason for ICU admission was associated with earlier death (aHR 2.14, 95% CI 1.04–4.38; *p* = 0.038), whereas respiratory failure was not (aHR 1.30, 95% CI 0.69–2.45; *p* = 0.419).

After addition of NLR, each doubling of NLR was independently associated with a higher hazard of in-hospital death (aHR 1.21, 95% CI 1.02–1.44; *p* = 0.026), while hepatic metastatic involvement remained independently associated with outcome (aHR 1.89, 95% CI 1.06–3.38; *p* = 0.031). In contrast, PLR and SII were not independently associated with time to death (aHR 0.96, 95% CI 0.81–1.13; *p* = 0.634 and aHR 1.01, 95% CI 0.91–1.12; *p* = 0.879, respectively). Higher platelet count was independently associated with a lower hazard of death, with each 50 × 10^9^/L increase corresponding to an aHR of 0.93 (95% CI 0.87–1.00; *p* = 0.047).

In the exploratory model jointly incorporating NLR and platelet count, both markers retained independent associations with time to death. Each doubling of NLR was associated with a higher hazard of death (aHR 1.28, 95% CI 1.08–1.53; *p* = 0.005), whereas each 50 × 10^9^/L increase in platelet count was associated with a lower hazard (aHR 0.91, 95% CI 0.84–0.98; *p* = 0.011). Hepatic metastatic involvement also remained independently associated with earlier death (aHR 1.85, 95% CI 1.03–3.33; *p* = 0.039). Neither age nor ICU admission phenotype reached statistical significance in the combined model.

No evidence of violation of the proportional-hazards assumption was observed across the multivariable models, with global Schoenfeld-test *p*-values ranging from 0.405 to 0.641.

In a sensitivity analysis retaining the original six-category classification of the primary reason for ICU admission, the associations of NLR and platelet count with time to death were essentially unchanged. NLR remained associated with a higher hazard of death (aHR 1.28, 95% CI 1.07–1.52; *p* = 0.006), whereas platelet count remained inversely associated (aHR 0.91, 95% CI 0.84–0.98; *p* = 0.010). Hepatic metastatic involvement also remained independently associated with time to death (aHR 1.90, 95% CI 1.04–3.46; *p* = 0.036). The global proportional-hazards assumption remained satisfied (*p* = 0.89).

Following additional adjustment for APACHE II, the combined Cox proportional-hazards model showed persistent associations between hepatic metastatic involvement, platelet count, NLR, and time to in-hospital death (Table 7). Hepatic metastatic involvement was associated with an increased hazard of death (aHR 2.06, 95% CI 1.14–3.74; *p* = 0.017), whereas higher platelet count was associated with a lower hazard (aHR 0.91 per 50 × 10^9^/L increase, 95% CI 0.84–0.98; *p* = 0.010). Each doubling of NLR was associated with a higher hazard of death (aHR 1.25, 95% CI 1.05–1.48; *p* = 0.011). APACHE II was also associated with the timing of death (aHR 1.28 per five-point increase, 95% CI 1.07–1.53; *p* = 0.006). However, the proportional-hazards assumption was violated globally (*p* = 0.038), primarily because of the APACHE II term (*p* = 0.003), indicating that a constant hazard ratio may not adequately describe the association between physiological severity and mortality throughout follow-up.

Complementary analyses were performed among patients who remained alive and hospitalized beyond 24 and 48 h after ICU admission (Table 8). Hepatic metastatic involvement remained associated with subsequent mortality at both landmarks, with adjusted hazard ratios of 1.87 (95% CI 1.03–3.40; *p* = 0.040) and 2.03 (95% CI 1.07–3.87; *p* = 0.031), respectively. Higher platelet count retained an inverse association with mortality at 24 h (aHR 0.88, 95% CI 0.81–0.96; *p* = 0.002) and 48 h (aHR 0.90, 95% CI 0.83–0.98; *p* = 0.015). NLR also remained associated with subsequent time to death at both landmarks (aHR 1.31, 95% CI 1.09–1.57; *p* = 0.003, and aHR 1.24, 95% CI 1.01–1.50; *p* = 0.036, respectively). Although the global proportional-hazards tests were not statistically significant, the APACHE II term demonstrated evidence of non-proportionality in the 24-h landmark model (*p* = 0.026), indicating cautious interpretation.

Internal validation using 1000 bootstrap resamples demonstrated moderate discrimination of the Core ICU model for binary in-hospital mortality (Table 9). The model achieved an apparent AUROC of 0.784 and an optimism-corrected AUROC of 0.743 (95% CI 0.654–0.860), with a corrected Brier score of 0.141 (95% CI 0.091–0.169) and a corrected calibration slope of 0.896. Addition of NLR resulted in only a minimal improvement in corrected discrimination (AUROC 0.745), whereas PLR and SII did not improve performance, yielding corrected AUROCs of 0.734 and 0.732, respectively.

The addition of baseline platelet count increased the optimism-corrected AUROC to 0.799 (95% CI 0.731–0.897), corresponding to an improvement of 0.056 (95% CI 0.002–0.146) over the Core ICU model. The corrected Brier score decreased to 0.134 (95% CI 0.086–0.160), while the corrected calibration slope was 0.867. The combined NLR–platelet model achieved the highest optimism-corrected AUROC of 0.802 (95% CI 0.741–0.902) and the lowest corrected Brier score of 0.131 (95% CI 0.083–0.156). However, the incremental improvement in discrimination following the addition of NLR to the platelet-containing model was minimal (ΔAUROC 0.004, 95% CI −0.012 to 0.044).

The optimism-corrected calibration slopes ranged from 0.841 to 0.896 across models, indicating some degree of overfitting. Although platelet count improved discrimination relative to the basic clinical model, uncertainty in the estimated differences and the absence of a demonstrable additional benefit from NLR warrant cautious interpretation. Detailed paired comparisons of optimism-corrected AUROC and Brier scores, together with approximate 95% confidence intervals, are provided in Supplementary Table S1. Calibration plots for the original clinical models are presented in Supplementary Figure S1.

Internal validation using 1000 bootstrap resamples demonstrated moderate discrimination of the severity-adjusted Core model, which incorporated APACHE II, hepatic metastatic involvement, and primary reason for ICU admission (Table 10). The model achieved an apparent AUROC of 0.787 and an optimism-corrected AUROC of 0.745, with a corrected Brier score of 0.141 and calibration slope of 0.899.

The addition of baseline platelet count improved model discrimination, increasing the optimism-corrected AUROC from 0.745 to 0.793 (ΔAUROC = 0.048). Concurrently, the corrected Brier score decreased from 0.141 to 0.134, indicating improved overall predictive accuracy. The corresponding optimism-corrected calibration slope was 0.882.

Further incorporation of NLR alongside platelet count did not produce additional improvement in discrimination, with the corrected AUROC decreasing to 0.786. Nevertheless, the combined model achieved the lowest corrected Brier score (0.132), suggesting a modest reduction in overall prediction error, while its corrected calibration slope was 0.858. The estimated improvement in discrimination following the addition of platelet count to the APACHE II-adjusted Core model was accompanied by considerable uncertainty (ΔAUROC 0.048, 95% CI −0.0003 to 0.128; Supplementary Table S1). Calibration plots for the APACHE II-adjusted models are presented in Supplementary Figure S2.

Decision-curve analysis was performed across threshold probabilities ranging from 0.50 to 0.85. Although the addition of platelet count or NLR yielded positive point estimates of incremental net benefit at selected thresholds, the approximate 95% confidence intervals for the between-model differences included zero at all reported thresholds (Supplementary Table S2). Decision curves for the original clinical models and APACHE II-adjusted models are presented in Supplementary Figures S3 and S4, respectively. These findings did not establish a consistent incremental clinical benefit. Given that the evaluated thresholds were not associated with predefined clinical interventions, the analyses were considered exploratory.

Sensitivity analyses excluding patients who died shortly after ICU admission yielded findings consistent with the primary analysis (Table 11). Hepatic metastatic involvement remained strongly associated with in-hospital mortality after exclusion of deaths occurring within 24 h (aOR 7.36, 95% CI 2.23–28.06; *p* < 0.001) and 48 h (aOR 7.12, 95% CI 2.14–27.18; *p* = 0.001), compared with an aOR of 8.01 (95% CI 2.42–30.75; *p* < 0.001) in the primary analysis. Associations with ICU admission phenotype were similarly stable. Respiratory failure remained associated with markedly higher odds of mortality after exclusion of deaths within 24 h (aOR 11.55, 95% CI 3.07–51.79; *p* < 0.001) and 48 h (aOR 10.63, 95% CI 2.84–47.20; *p* < 0.001), as did other critical illness.

The inverse association between platelet count and mortality was also highly consistent across sensitivity analyses. The aOR per 50 × 10^9^/L increase was 0.79 (95% CI 0.66–0.92; *p* = 0.002) in the primary analysis, 0.79 (95% CI 0.66–0.92; *p* = 0.002) after exclusion of deaths within 24 h, and 0.81 (95% CI 0.68–0.94; *p* = 0.006) after exclusion of deaths within 48 h. NLR retained a positive but non-significant association with binary mortality, with aORs of 1.39, 1.36, and 1.31 per doubling across the three analyses, respectively. Age was not independently associated with mortality in any model.

Additional sensitivity analyses incorporating APACHE II demonstrated consistent associations after exclusion of very early in-hospital deaths (Table 12). Hepatic metastatic involvement remained significantly associated with mortality after exclusion of deaths within 24 h (aOR 7.01, 95% CI 2.14–25.99; *p* = 0.0012) and 48 h (aOR 6.74, 95% CI 2.06–24.86; *p* = 0.0015). The inverse association between platelet count and mortality also persisted, with corresponding aORs of 0.81 (95% CI 0.68–0.94; *p* = 0.004) and 0.83 (95% CI 0.70–0.96; *p* = 0.011). Neither APACHE II nor NLR was significantly associated with binary mortality in the restricted cohorts.

Longitudinal analyses demonstrated distinct biological trajectories between survivors and non-survivors (Table 13). Baseline creatinine, urea, and INR were comparable between outcome groups (all *p* > 0.05), whereas baseline AST was higher among non-survivors (45.00 [24.00–93.00] vs. 27.50 [19.75–33.50]; *p* = 0.016). Differences became substantially more pronounced at the final post-admission assessment, with final creatinine, urea, INR, and AST all significantly higher among non-survivors (all *p* < 0.001).

Baseline-to-final changes consistently indicated greater biological deterioration among non-survivors. Creatinine increased by a median of 0.42 [−0.02–1.01] among non-survivors, whereas survivors showed a median decrease of 0.14 [−0.41–0.00] (*p* < 0.001). Urea increased by 28.00 [−1.00–66.25] among non-survivors compared with 0.00 [−16.00–12.75] among survivors (*p* = 0.002).

Greater increases were also observed for INR (0.15 [−0.02–0.52] vs. −0.07 [−0.12–0.27]; *p* = 0.041) and AST (6.50 [−12.25–91.00] vs. −1.50 [−15.25–0.50]; *p* = 0.012). All four differences in biological change remained significant after Benjamini–Hochberg correction, with FDR-adjusted *p*-values of <0.001, 0.005, 0.041, and 0.016 for creatinine, urea, INR, and AST, respectively.

The recalculated inflammation-based indices showed moderate-to-strong positive intercorrelations (Table 14). The strongest correlation was observed between NLR and SII (Spearman ρ = 0.804; *p* < 0.001), while PLR was also strongly correlated with SII (ρ = 0.686; *p* < 0.001). The correlation between NLR and PLR was moderate (ρ = 0.517; *p* < 0.001). These findings indicate substantial, although incomplete, overlap among the three inflammation-based indices.).

Competing-risk analysis demonstrated a rapid accumulation of in-hospital deaths following ICU admission (Table 15). The cumulative incidence of death was 5.0% (95% CI 1.1–8.9%) at day 1 and increased to 22.5% (95% CI 15.0–30.0%) by day 3. By day 7, nearly half of the cohort had experienced in-hospital death (48.3%, 95% CI 39.3–57.3%), with cumulative incidence increasing to 66.7% (95% CI 58.1–75.2%) at day 14 and 77.5% (95% CI 69.9–85.1%) at day 28.

The competing cumulative incidence of discharge alive was substantially lower, reaching 7.5% (95% CI 2.8–12.2%) at day 7, 13.3% (95% CI 7.2–19.5%) at day 14, and 18.3% (95% CI 11.3–25.4%) at day 28.

Fine–Gray competing-risk regression yielded findings consistent with the cause-specific Cox analysis after additional adjustment for primary reason for ICU admission (Table 16). Hepatic metastatic involvement remained independently associated with the cumulative incidence of in-hospital death (sHR 2.33, 95% CI 1.37–3.97; *p* = 0.002). Age was not independently associated with the subdistribution hazard of death (sHR 1.19 per 10-year increase, 95% CI 0.99–1.43; *p* = 0.065). Compared with neurological deterioration, respiratory failure showed an elevated but non-significant subdistribution hazard (sHR 2.02, 95% CI 0.98–4.18; *p* = 0.058), while the estimate for other critical illness was of borderline statistical significance (sHR 2.32, 95% CI 1.00–5.39; *p* = 0.050).

NLR and platelet count retained independent and opposing associations with the cumulative incidence of death. Each doubling of NLR was associated with a 25% higher subdistribution hazard of in-hospital death (sHR 1.25, 95% CI 1.07–1.45; *p* = 0.004), whereas each 50 × 10^9^/L increase in platelet count was associated with a 10% lower subdistribution hazard (sHR 0.90, 95% CI 0.84–0.97; *p* = 0.003).

Additional Fine–Gray competing-risk regression incorporating APACHE II yielded associations consistent in direction with the original analysis (Table 17). Hepatic metastatic involvement remained associated with a higher subdistribution hazard of in-hospital death (sHR 2.70, 95% CI 1.46–4.98; *p* = 0.001). Higher APACHE II scores were also associated with the cumulative incidence of death (sHR 1.32 per five-point increase, 95% CI 1.10–1.58; *p* = 0.003). Compared with neurological deterioration, both respiratory failure (sHR 2.37, 95% CI 1.09–5.12; *p* = 0.029) and other critical illness (sHR 2.67, 95% CI 1.13–6.31; *p* = 0.025) were associated with higher subdistribution hazards of death. Among hematological predictors, each doubling of NLR was associated with a higher subdistribution hazard (sHR 1.20, 95% CI 1.03–1.40; *p* = 0.018), whereas each 50 × 10^9^/L increase in platelet count was associated with a lower subdistribution hazard (sHR 0.90, 95% CI 0.84–0.97; *p* = 0.008).

Additional Fine–Gray analyses supported the persistence of the observed prognostic associations after the initial 24 and 48 h of hospitalization (Table 18). Hepatic metastatic involvement remained associated with the cumulative incidence of subsequent in-hospital death at both landmarks (sHR 2.41, 95% CI 1.33–4.37; *p* = 0.004, and sHR 2.31, 95% CI 1.24–4.29; *p* = 0.008, respectively). Higher platelet count was consistently associated with a lower subdistribution hazard of death (sHR 0.88, 95% CI 0.82–0.95; *p* = 0.001 at 24 h, and sHR 0.90, 95% CI 0.83–0.97; *p* = 0.008 at 48 h). NLR retained a positive association at both landmarks (sHR 1.24, 95% CI 1.05–1.47; *p* = 0.013, and sHR 1.18, 95% CI 1.004–1.396; *p* = 0.044, respectively). APACHE II was associated with the subdistribution hazard of death at the 24-h landmark (sHR 1.23, 95% CI 1.02–1.48; *p* = 0.033), whereas the corresponding association at 48 h did not reach statistical significance (*p* = 0.090).

**Figure 3:**
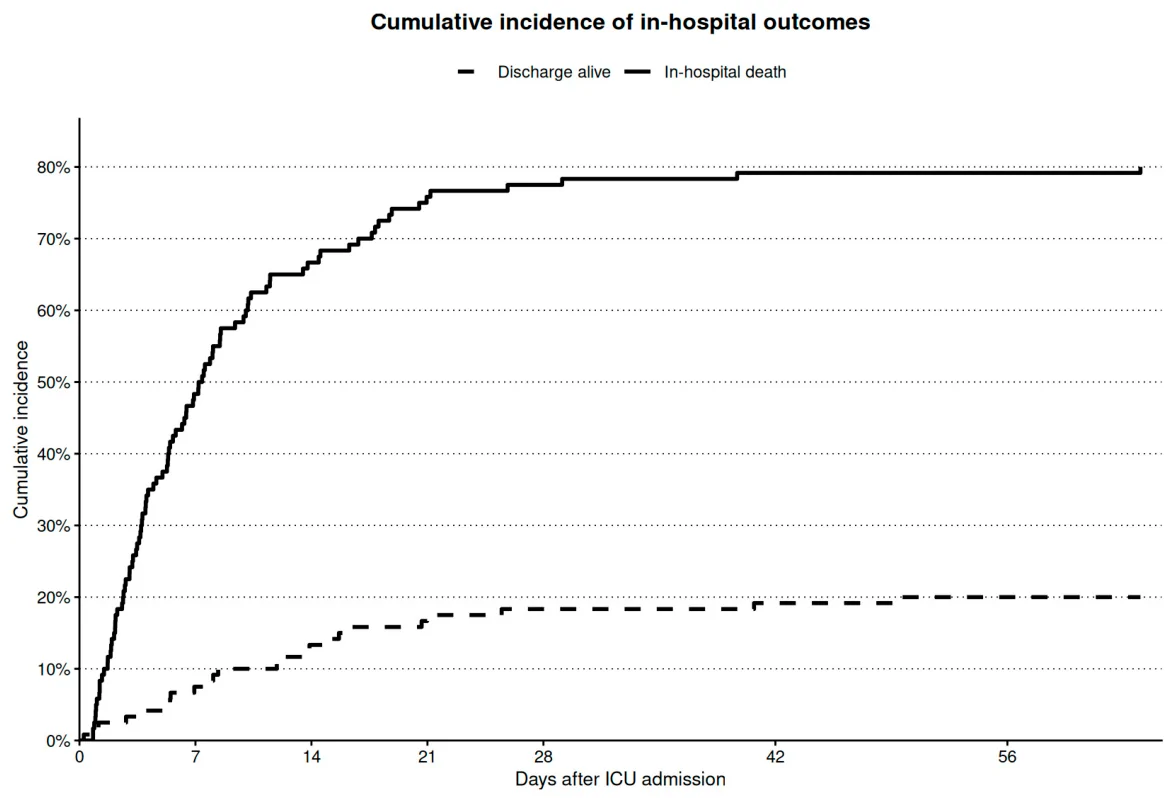
Cumulative incidence of in-hospital death and discharge alive following ICU admission.

The cumulative incidence curves further illustrated the rapid accumulation of in-hospital deaths during the early period following ICU admission (Figure 3). The incidence of death increased steeply during the first two weeks, reaching 48.3% by day 7 and 66.7% by day 14, before increasing more gradually to 77.5% by day 28. On the other hand, discharge alive accumulated substantially more slowly throughout follow-up, with a cumulative incidence of 7.5% at day 7, 13.3% at day 14, and 18.3% at day 28.

## 4. Discussion

The present study provides several complementary insights into short-term prognosis among critically ill patients with neoplasia. In-hospital mortality was high (80.0%) and occurred early, with the cumulative incidence of death reaching 48.3% by day 7 and 77.5% by day 28 when discharge alive was considered as a competing event. Hepatic metastatic involvement and the clinical presentation leading to ICU admission were strongly associated with the occurrence of in-hospital death. These associations remained robust after adjustment for routinely available hematological markers and after exclusion of very early deaths.

The prognostic information provided by hematological markers differed according to the outcome framework. Higher platelet count was independently associated with lower odds of in-hospital mortality and improved internally validated model performance, whereas NLR was not independently associated with binary mortality. In time-to-event analyses, however, higher NLR and lower platelet count were independently associated with earlier death, and these associations remained consistent when discharge alive was treated as a competing event. Together, these findings suggest that oncological burden, acute clinical presentation, hematological status, and subsequent organ dysfunction capture complementary dimensions of short-term prognosis.

The observed 80% in-hospital mortality was substantially higher than that reported in most contemporary ICU oncology cohorts. In the 2025 scoping review by Codru and Vecerzan, 73 studies encompassing more than 170,000 ICU admissions across 27 countries were synthesized. Weighted ICU and hospital mortality were approximately 41% and 38%, respectively, although mortality varied widely according to case mix, cancer status, organ-support burden, and reversibility of acute illness [2]. The cited review further suggested that benefit from intensive care was concentrated among patients admitted early, those with limited organ failure, controlled or potentially treatable malignancy, and reversible treatment-related complications, whereas very high organ-support requirements and refractory or progressive disease were associated with markedly poorer outcomes.

Large contemporary cohorts support the same degree of heterogeneity. Vigneron et al. retrospectively evaluated 1525 adults with solid malignancies undergoing unplanned ICU admission and reported an ICU survival rate of 77.4% [1]. Metastatic disease remained independently associated with ICU mortality, with a cause-specific HR of 1.78 (95% CI 1.38–2.30). Díaz-Lagares et al., in a retrospective cohort of 395 consecutive patients with solid tumors undergoing unplanned ICU admission, reported ICU and in-hospital mortality rates of 27.9% and 39.0%, respectively [3]. Similarly, Wei et al. prospectively studied 1488 patients after unplanned ICU admission and observed 90-day mortality of 27.9% [17]. Worse prognosis was associated with higher acute illness severity, shock, respiratory failure, invasive ventilation, acute kidney injury, and the setting from which patients were transferred to the ICU [17].

More recent data reinforce the importance of both cancer burden and acute physiological derangement. Badheeb et al. reported ICU mortality of 38.1% among 105 patients with active solid or hematologic malignancies, with older age, advanced-stage disease, respiratory infection, and higher SOFA scores independently associated with mortality [18]. Advanced-stage disease carried an adjusted OR of 3.37 (95% CI 1.56–7.26), while higher SOFA scores were associated with an aOR of 1.70 per point [18].

Bosch-Compte et al. similarly reported hospital mortality of 40.3% among 129 consecutive patients with solid tumors admitted to a medical ICU [8]. Metastatic disease, neutropenia, greater organ dysfunction, and invasive mechanical ventilation were among the independent adverse factors, with metastatic disease associated with an HR of 3.92 (95% CI 1.82–8.45) [8].

Even in cohorts restricted to metastatic cancer, mortality remains highly variable. Oud analyzed 136,644 ICU admissions involving patients with metastatic malignancy and found overall short-term mortality of 28.1%, rising to 62.0% among mechanically ventilated patients [19]. Increasing organ-failure burden was independently associated with mortality. This literature data indicates that metastatic disease alone is insufficient to explain outcome and that the interaction between cancer burden and acute physiological failure is critical.

This concept is further supported by recent machine-learning data. Nieto et al. analyzed 997 critically ill patients with cancer using 46 demographic, oncologic, physiological, laboratory, and therapeutic variables and found that short-term ICU mortality was driven predominantly by acute physiological derangements rather than by cancer diagnosis alone [20]. Lactate, platelet count, blood urea nitrogen, vasopressor-related variables, and global severity measures were among the most influential predictors, while 30-day survival incorporated a broader contribution from baseline physiological status, age, creatinine, and other post-ICU determinants [20]. Similarly, Wang et al. analyzed 1739 critically ill patients with malignancy from MIMIC-IV and identified BUN, albumin, respiratory rate, heart rate, and SOFA among the principal predictors of short-term mortality [21].

Our cohort represented a particularly high-risk ICU population. Nearly all patients had metastatic disease (95.8%), approximately four fifths had hepatic metastatic involvement, and cachexia was frequent. Organ-support requirements during the ICU course were also significant: 79.2% of patients required vasopressors and 58.3% underwent invasive mechanical ventilation. Both interventions were markedly more frequent among non-survivors. Although these treatment variables were not incorporated into the baseline prognostic models because they could occur after ICU admission, they illustrate the severity of the subsequent clinical course and help contextualize the observed high in-hospital mortality rate.

The markedly higher mortality observed in our cohort compared with most contemporary ICU oncology series should be interpreted in the context of this highly selected case mix. As a retrospective single-center study, the present analysis cannot determine to what extent the observed mortality reflects differences in local ICU referral and admission practices, timing of ICU transfer, disease trajectory before admission, or the availability of potentially reversible oncological treatment options. Detailed information on ICU triage decisions, patients who were considered but not admitted to the ICU, and the timing of clinical deterioration relative to ICU referral was not systematically available. Consequently, selection toward patients with more advanced malignancy or more severe acute deterioration cannot be excluded and may have contributed to the unusually high mortality observed in this cohort.

Hepatic metastatic involvement showed a consistent association with in-hospital mortality across the evaluated models. Liver metastases were present in 85.4% of non-survivors compared with 54.2% of survivors and remained associated with mortality after adjustment for age and primary reason for ICU admission. In the Core ICU model, hepatic metastatic involvement was associated with an aOR of 6.96 (95% CI 2.41–21.80), while in the exploratory combined NLR–platelet model the corresponding aOR was 8.01 (95% CI 2.42–30.80). Although the direction and statistical significance of this association were consistent across model specifications, the wide confidence intervals indicate substantial uncertainty regarding its magnitude. Therefore, these estimates should be interpreted primarily as evidence of an association rather than as precise measures of effect size, particularly given the limited number of survivors. Also, hepatic metastases may reflect greater overall metastatic burden, differences in primary tumor type or disease biology, and impaired hepatic reserve or dysfunction.

The adverse prognostic significance of metastatic disease in general is well established in ICU oncology. Vigneron et al. [1], Bosch-Compte et al. [8], Badheeb et al., [18], and the meta-analysis by Elkhapery et al. [22], all support an association between advanced or metastatic disease and mortality. Elkhapery et al. synthesized 33 studies of critically ill patients with lung cancer, including 28 cohort studies and five population-based studies, and reported pooled ICU and hospital mortality of 46.4% and 51.9%, respectively [22]. Metastatic disease was associated with increased mortality with a pooled RR of 1.30 (95% CI 1.06–1.59), alongside poor performance status, mechanical ventilation, vasopressor requirement, and higher severity scores.

However, most ICU studies characterize oncologic burden using cancer stage or metastatic status rather than the anatomical distribution of metastases. This distinction is important because 115 of 120 patients in our cohort were already metastatic, making a simple metastatic/non-metastatic dichotomy intrinsically less informative. In such a population, site-specific metastatic involvement may provide additional clinical separation.

The study by García de Herreros et al. represents the closest ICU comparator. Their retrospective study included 97 ICU admissions involving 84 patients with solid malignancies, 79.5% of whom had metastatic disease [9]. Liver metastases were among the variables associated with greater mortality, but hepatic involvement was not retained in the final adjusted model. Instead, ECOG performance status >2, basal CRP >0.5 mg/dL, and admission from a hospital ward remained independently associated with in-hospital mortality. Their in-hospital mortality was also substantially lower than ours, at 24% [9]. Our findings therefore extend rather than replicate this observation, as hepatic involvement remained strongly associated with mortality after penalized adjustment and across multiple model specifications.

Broader oncologic evidence also supports the adverse prognostic relevance of hepatic metastatic involvement. Waninger et al. conducted a systematic review and meta-analysis of 83 studies including 604,853 patients and reported a pooled overall-survival HR of 1.77 (95% CI 1.62–1.93) for patients with liver metastases [23]. Tian et al., in a meta-analysis of 163 studies of patients receiving immune checkpoint inhibitors, found liver metastases associated with poorer overall survival (HR 1.82, 95% CI 1.59–2.08) and progression-free survival (HR 1.68, 95% CI 1.49–1.89) [24].

The ARCAD pooled analysis provides further evidence that hepatic involvement may represent a distinct prognostic dimension. Cohen et al. analyzed 17,924 patients with metastatic colorectal cancer from 26 randomized trials, of whom 14,066 had liver metastases [25]. Across several chemotherapy and anti-VEGF treatment settings, patients without liver metastases experienced superior OS and PFS, although the effect varied by treatment line and regimen. The prognostic effect was not uniform in anti-EGFR-treated populations, indicating that hepatic involvement should not be regarded as an invariant biological determinant independent of clinical context.

The persistence of the liver-metastasis effect after exclusion of very early deaths is particularly relevant. In the combined model, the aOR for hepatic metastatic involvement was 8.01 in the full cohort, 7.36 after exclusion of deaths within 24 h, and 7.12 after exclusion of deaths within 48 h. The similar direction of the estimates across these sensitivity analyses suggests that the observed association was not driven exclusively by patients admitted in an immediately preterminal condition. However, the confidence intervals remained wide, and the limited number of survivors restricts the precision with which the magnitude of this association can be estimated. These findings cannot establish causality. Liver metastases may instead function as a marker of greater systemic tumor burden, aggressive disease biology, impaired hepatic reserve, altered metabolism, or other unmeasured characteristics associated with poor reversibility during acute illness. Thus, the consistency of the association across model specifications and sensitivity analyses supports further evaluation of metastatic pattern rather than metastatic status alone in larger ICU oncology cohorts.

The findings for inflammation-based indices were more nuanced. NLR, PLR, and SII were not independently associated with binary in-hospital mortality after adjustment for age, hepatic metastatic involvement, and ICU admission phenotype. However, NLR behaved differently when the timing of death was considered.

The prognostic relevance of NLR in general ICU populations has been well described. Salciccioli et al. evaluated 5056 unselected ICU patients and found a stepwise increase in 28-day mortality across increasing NLR quartiles after multivariable adjustment [26]. The highest quartile was associated with an OR of 1.71 (95% CI 1.35–2.16) compared with the lowest. However, this association was absent among patients with sepsis, already suggesting that the meaning of NLR varies according to the dominant inflammatory context. Buonacera et al. similarly emphasized NLR as a marker reflecting the balance between innate inflammatory activation and adaptive immune competence across cancer, infection, and critical illness [27].

Tunay et al. retrospectively studied 229 patients with lung cancer admitted to intensive care and evaluated NLR, PLR, LMR, SII, modified Glasgow prognostic score, PNI, APACHE II, and MPM II-Admission [14]. Although several indices displayed univariable or ROC signals, multivariable analysis identified PNI as the only inflammation-related parameter independently associated with mortality. NLR, PLR, LMR, and SII were not retained as independent predictors.

The performance of inflammatory indices in other ICU populations illustrates how strongly context modifies their prognostic value. In 267 patients with blood-culture-confirmed sepsis, Mangalesh et al. found SII, NLR, and PLR independently associated with mortality, with adjusted ORs of 1.51, 1.67, and 1.24, respectively [28]. SII achieved an AUROC of 0.848 and improved prognostic performance when added to SOFA, with significant NRI and IDI. Zhao et al., in 869 critically ill patients with intracerebral hemorrhage, found NLR to provide the best discrimination among NLR, PLR, LMR, SII, and SIRI, although standalone discrimination remained moderate [10]. Zhou et al. similarly incorporated NLR and LMR into a multivariable model for 28-day mortality among ICU patients with acute pulmonary embolism, achieving an AUROC of 0.772 [29].

These contrasting findings support the interpretation that inflammation-based indices are context-dependent rather than universally predictive. In critically ill patients with cancer, leukocyte and platelet counts may simultaneously reflect tumor-associated inflammation, acute infection, physiological stress, corticosteroid exposure, recent anticancer therapy, marrow suppression, transfusion, and evolving organ dysfunction. The resulting inflammatory signal is therefore biologically heterogeneous.

The influence of infection on these indices is particularly relevant. Wang et al. studied 417 hospitalized patients with lung cancer and found that those with concomitant pulmonary infection had lower PNI and higher SII than patients without infection [30]. Lower PNI and higher SII were independently associated with infection, while among infected patients lower PNI and higher MLR were independently associated with in-hospital mortality. Their combined model achieved an AUROC of 0.806 [30]. These findings demonstrate that inflammatory and nutritional biomarkers in cancer may be materially altered by acute infectious processes and should not be interpreted as direct surrogates of tumor-associated inflammation.

NLR was not independently associated with the occurrence of in-hospital death after adjustment for age, hepatic metastatic involvement, and ICU admission phenotype (aOR 1.21 per doubling, 95% CI 0.87–1.75; *p* = 0.264). In the combined NLR–platelet model, the association remained non-significant (aOR 1.39, 95% CI 0.96–2.07; *p* = 0.080), with similar estimates after exclusion of deaths within 24 and 48 h. In contrast, each doubling of NLR was independently associated with a higher hazard of death in the Cox model (aHR 1.21, 95% CI 1.02–1.44; *p* = 0.026), with a stronger association when NLR and platelet count were included simultaneously (aHR 1.28, 95% CI 1.08–1.53; *p* = 0.005). Fine–Gray regression yielded a consistent estimate after accounting for discharge alive as a competing event (sHR 1.25, 95% CI 1.07–1.45; *p* = 0.004). These findings should not be considered contradictory. Logistic regression addresses whether death occurs, whereas time-to-event analyses additionally incorporate when it occurs. In a cohort in which 80% of patients experienced the primary endpoint, NLR may therefore provide greater information on the timing of fatal deterioration than on binary outcome classification.

Evidence from other critically ill populations supports the concept that dynamic or survival-oriented analyses may capture aspects of inflammatory burden that are not apparent in a simple binary endpoint. Dongqing et al. studied 3350 critically ill children and found higher time-weighted NLR independently associated with increased in-hospital mortality (OR 1.15, 95% CI 1.08–1.22), while Kaplan–Meier analysis showed significant differences in 90-day survival [31]. Although this pediatric population is not directly comparable to adult ICU oncology, it supports the broader principle that temporal inflammatory exposure may relate to the trajectory of critical illness.

The model-comparison results reinforce the distinction between prognostic association and incremental prediction. Wu et al. analyzed 21,822 ICU patients from MIMIC-III and found that NLR alone had limited discrimination for mortality, with an AUC of 0.609 [32]. Adding NLR to SAPS II increased AUC from 0.789 to 0.798, a statistically measurable but modest improvement. In oncology, Rocco et al. analyzed 2286 patients with hepatocellular carcinoma and found NLR and PLR independently associated with OS [33]. Their combined NLR–PLR measure produced a statistically significant but small incremental improvement when added to a clinical prognostic model, with an IDI of 1.3%.

Platelet count emerged as a complementary hematological marker with a different prognostic profile. After adjustment for age, hepatic metastatic involvement, and ICU admission phenotype, each 50 × 10^9^/L increase in platelet count was associated with lower odds of in-hospital mortality (aOR 0.81, 95% CI 0.67–0.94; *p* = 0.006). The association was even more pronounced in the combined NLR–platelet model (aOR 0.79, 95% CI 0.66–0.92; *p* = 0.002) and remained remarkably stable after exclusion of deaths within 24 h (aOR 0.79) and 48 h (aOR 0.81). Higher platelet count was also associated with a lower hazard of death in the Cox model (aHR 0.93, 95% CI 0.87–1.00; *p* = 0.047) and remained independently associated when modeled jointly with NLR (aHR 0.91, 95% CI 0.84–0.98; *p* = 0.011). Fine–Gray regression yielded a consistent estimate after accounting for discharge alive (sHR 0.90, 95% CI 0.84–0.97; *p* = 0.003).

In critically ill patients with advanced malignancy, lower platelet counts may integrate several processes, including marrow suppression, recent anticancer treatment, infection, consumption, hepatic dysfunction, and greater systemic illness severity. Therefore, these markers should be interpreted as indicators of oncological and critical-illness processes rather than specific measures of tumor-associated inflammation. The identification of platelet count as an informative variable is also consistent with the machine-learning study by Nieto et al., in which platelet count was among the influential predictors of short-term ICU mortality [20].

The model-performance analyses further highlighted the distinction between prognostic association and incremental predictive value. The original Core ICU model achieved an optimism-corrected AUROC of 0.743, whereas the addition of platelet count increased discrimination to 0.799 (ΔAUROC 0.056, 95% CI 0.002–0.146). The combined NLR–platelet model achieved the highest corrected AUROC of 0.802; however, the additional improvement over the platelet-containing model was negligible (ΔAUROC 0.004, 95% CI −0.012 to 0.044). Neither NLR alone nor PLR or SII demonstrated a meaningful improvement in discrimination. Following adjustment for APACHE II, the addition of platelet count was associated with an increase in corrected AUROC from 0.745 to 0.793, although the corresponding confidence interval for the difference included zero (ΔAUROC 0.048, 95% CI −0.0003 to 0.128). Also, none of the pairwise differences in corrected Brier scores demonstrated a confidence interval excluding zero. These findings suggest that platelet count may contribute incremental predictive information beyond the clinical model, whereas the magnitude and reproducibility of this contribution become less certain after adjustment for physiological severity. The addition of NLR does not appear to provide a substantial further improvement in binary mortality discrimination. Thus, the apparent incremental performance of the combined model requires external validation in a larger cohort.

Optimism-corrected calibration slopes ranged from 0.841 to 0.899 across the evaluated models, remaining consistently below the ideal value of 1. This pattern suggests that predicted probabilities may be excessively dispersed, with some residual overfitting despite penalized estimation and bootstrap internal validation. Although calibration-in-the-large estimates indicated relatively small deviations in overall predicted risk, these findings do not establish adequate calibration across the entire risk spectrum, particularly in a cohort characterized by a high mortality rate and a limited number of survivors. The decision-curve analysis also failed to demonstrate a consistent incremental net benefit from adding hematological predictors.

After adjustment for APACHE II, hepatic metastatic involvement (aOR 7.87, 95% CI 2.41–29.44) and platelet count (aOR 0.82, 95% CI 0.69–0.94) retained significant associations with binary in-hospital mortality, whereas neither NLR nor APACHE II was statistically significant in the combined Firth model. On the other hand, the APACHE II-adjusted Fine–Gray analysis demonstrated significant associations of physiological severity, hepatic metastatic involvement, NLR, and platelet count with the cumulative incidence of death.

Post-admission biological evolution provided a separate perspective on the fatal trajectory. Baseline creatinine, urea, and INR were similar between survivors and non-survivors, whereas their final post-admission values were significantly higher among patients who died. More importantly, baseline-to-final changes differed between outcome groups: non-survivors showed greater increases in creatinine, urea, INR, and AST, and all four differences remained significant after false-discovery-rate correction. These findings suggest that progressive renal, coagulation, and hepatic abnormalities characterize the evolving fatal course rather than constituting baseline predictors of mortality.

The distinction between baseline risk and dynamic organ dysfunction is well supported by literature data. Díaz-Lagares et al. found that a non-decreasing SOFA score by day 5 was associated with adverse prognosis, emphasizing that failure of organ dysfunction to improve provides information beyond admission status alone [3]. Azoulay et al., in the Intensive Care Medicine research agenda for critically ill oncology and hematology patients, similarly identified improved characterization of organ dysfunction, reversibility, and timing of intensive-care interventions as major research priorities [34].

The renal findings are particularly consistent with recent ICU oncology data. Nieto et al. identified blood urea nitrogen among the most influential predictors of ICU mortality and creatinine among important predictors of 30-day survival [20]. Wang et al. likewise identified BUN and albumin among the strongest predictors of 14- and 28-day mortality [21]. The recent scoping review and meta-analysis by Paulus et al. further provides a mechanistic framework for interpreting urea-related abnormalities [35]. Across 47 critical-care studies, an elevated urea-to-creatinine ratio was associated with persistent catabolic stress and adverse outcomes. The authors, however, emphasized that dehydration, gastrointestinal bleeding, kidney dysfunction, liver dysfunction, and renal replacement therapy can all influence this marker.

From a clinical perspective, the findings support a multidimensional view of short-term prognosis rather than reliance on a single biomarker. Hepatic metastatic involvement and the clinical presentation leading to ICU admission provided strong baseline information regarding the occurrence of in-hospital death. Platelet count contributed additional information to binary mortality prediction, whereas NLR was more informative regarding the timing of fatal deterioration. When evaluated jointly, NLR and platelet count remained independently associated with time to death in both Cox and Fine–Gray analyses and produced the highest internally validated discrimination for binary mortality. Post-admission increases in creatinine, urea, INR, and AST further characterized the fatal trajectory but represent evolving organ dysfunction rather than baseline prognostic information.

This study has important limitations. The retrospective design precludes causal inference and remains susceptible to residual confounding and incomplete measurement. The analyzed cohort included only 24 survivors, limiting the effective information available for multivariable modeling. Consequently, the number of covariate parameters in the extended models was high relative to the minority outcome group and may increase the risk of model overfitting and unstable coefficient estimates. Firth penalized-likelihood regression was used to reduce small-sample and separation-related bias, and bootstrap optimism correction was applied to model-performance estimates, but these approaches cannot fully overcome the limitations imposed by the small effective sample size. Thus, the magnitude of these associations should be interpreted cautiously. External validation in larger, preferably multicenter cohorts with a greater number of survivors is required.

The high mortality rates and metastatic burden indicate a highly selected population and restrict generalizability to other ICU oncology cohorts. Moreover, because detailed information regarding ICU referral and triage decisions, timing of transfer relative to clinical deterioration, and goals-of-care or treatment-limitation decisions was not systematically available, we cannot determine the extent to which local admission practices or end-of-life care patterns contributed to the observed mortality.

Tumor types were heterogeneous, while infection, corticosteroid exposure, recent anticancer therapy, marrow suppression, transfusion, and other factors may have influenced inflammatory indices. In addition, these covariates were not incorporated as covariates in the prognostic models and may have influenced NLR and platelet measurements. Residual confounding from these factors therefore cannot be excluded.

The management of oncological patients in critical state is often difficult and it is per-formed by a multidisciplinary team. Identifying clinical and biological factors with prognostic value is essential in the management of critically disease patients with neoplasms, facilitating a personalized approach and a more accurate estimation of mortality risk [36,37].

## 5. Conclusions

In this cohort of critically ill patients with neoplasia, hepatic metastatic involvement and the clinical presentation leading to ICU admission were associated with in-hospital mortality.

Higher platelet count was independently associated with lower mortality and improved internally validated discrimination when added to the core clinical model. NLR was not independently associated with binary mortality but was associated with earlier death, while platelet count retained an inverse association across binary, Cox, and competing-risk analyses.

The combined NLR–platelet model provided the highest internally validated performance, suggesting complementary prognostic information from these routinely available hematological markers.

Post-admission deterioration in renal, coagulation, and hepatic parameters further characterized the fatal clinical course.

External validation in larger, multicenter cohorts incorporating standardized measures of acute physiological severity is required before these findings can inform clinical prognostic models.

## Figures and Tables

**Table 1:** Baseline characteristics of the evaluated cohort.

Characteristic	Overall Cohort	Survivors	Non-Survivors	*p*-Value
Age, years	66.0 [59.0–73.0]	63.0 [54.8–73.2]	67.0 [60.0–73.0]	0.174
Female sex	43 (36.1%)	8 (33.3%)	35 (36.8%)	0.816
Primary tumor site				
Breast	11 (9.2%)	1 (4.2%)	10 (10.4%)	0.463 *
Cervical	5 (4.2%)	2 (8.3%)	3 (3.1%)	
Colorectal	27 (22.5%)	5 (20.8%)	22 (22.9%)	
Gastric	4 (3.3%)	1 (4.2%)	3 (3.1%)	
Head and neck	9 (7.5%)	3 (12.5%)	6 (6.3%)	
Hepatobiliary	3 (2.5%)	0 (0.0%)	3 (3.1%)	
Neuro-oncological	3 (2.5%)	2 (8.3%)	1 (1.0%)	
Other	5 (4.2%)	2 (8.3%)	3 (3.1%)	
Ovarian	4 (3.3%)	1 (4.2%)	3 (3.1%)	
Pancreatic	7 (5.8%)	2 (8.3%)	5 (5.2%)	
Pulmonary	25 (20.8%)	4 (16.7%)	21 (21.9%)	
Unknown primary	4 (3.3%)	0 (0.0%)	4 (4.2%)	
Urologic	12 (10.0%)	1 (4.2%)	11 (11.5%)	
Uterine	1 (0.8%)	0 (0.0%)	1 (1.0%)	
Metastatic disease	115 (95.8%)	21 (87.5%)	94 (97.9%)	0.054
Liver metastases	95 (79.2%)	13 (54.2%)	82 (85.4%)	0.002
Cachexia	65 (54.2%)	8 (33.3%)	57 (59.4%)	0.038
HGB, g/dL	10.1 [8.10–12.2]	9.25 [7.18–12.8]	10.1 [8.57–12.2]	0.646
WBC, ×10^9^/L	11.5 [6.74–18.2]	12.6 [7.74–16.3]	11.2 [6.74–18.6]	0.976
Platelet count, ×10^9^/L	226 [147–365]	274 [213–424]	218 [136–352]	0.066
Creatinine	1.42 [0.96–1.78]	1.38 [1.02–1.69]	1.42 [0.94–1.84]	0.798
Urea	70.5 [40.0–105]	68.0 [35.5–77.5]	72.5 [41.5–110]	0.180
NLR	8.97 [5.34–15.4]	8.13 [3.15–12.6]	9.17 [5.83–17.1]	0.165
PLR	217 [118–384]	242 [121–323]	217 [118–401]	0.966
SII	2159 [827–4376]	2458 [944–3392]	1966 [827–4472]	0.750
Vasopressor requirement	95/120 (79.2%)	5/24 (20.8%)	90/96 (93.8%)	<0.001
Invasive mechanical ventilation	70/120 (58.3%)	2/24 (8.3%)	68/96 (70.8%)	<0.001
Non-invasive ventilation	2/120 (1.7%)	0/24 (0.0%)	2/96 (2.1%)	1.000
Antibiotic therapy	108/120 (90.0%)	20/24 (83.3%)	88/96 (91.7%)	0.255
Corticosteroid therapy	67/120 (55.8%)	17/24 (70.8%)	50/96 (52.1%)	0.113
APACHE II	20.0 [14.75–24.25]	19.5 [14.50–22.25]	20.0 [14.75–25.00]	0.347

Legend: HGB, hemoglobin; WBC, white blood cell count; NLR, neutrophil-to-lymphocyte ratio; PLR, platelet-to-lymphocyte ratio; SII, systemic immune-inflammation index; IQR, interquartile range. * The *p*-value for primary tumor site represents the global Fisher’s exact test across all tumor-site categories.

**Table 2:** Univariable Firth penalized logistic regression analysis for in-hospital mortality.

Predictor	OR (95% CI)	*p*-Value
Age, per 10-year increase	1.35 (0.89–2.06)	0.155
Liver metastases	4.85 (1.85–12.89)	0.002
HGB, per 1 g/dL increase	1.04 (0.89–1.21)	0.657
WBC, per 1 × 10^9^/L increase	1.02 (0.98–1.07)	0.385
Platelet count, per 50 × 10^9^/L increase	0.88 (0.77–1.00)	0.045
Creatinine, per unit increase	1.10 (0.78–1.96)	0.644
Urea, per unit increase	1.01 (1.00–1.02)	0.131
NLR, per doubling	1.33 (0.98–1.82)	0.063
PLR, per doubling	1.05 (0.77–1.40)	0.752
SII, per doubling	1.07 (0.88–1.29)	0.486
Respiratory failure vs. neurological deterioration	4.60 (1.61–13.50)	0.004
Other critical illness vs. neurological deterioration	5.13 (1.48–20.40)	0.009

Legend: CI, confidence interval; HGB, hemoglobin; NLR, neutrophil-to-lymphocyte ratio; OR, odds ratio; PLR, platelet-to-lymphocyte ratio; SII, systemic immune-inflammation index; WBC, white blood cell count. NLR, PLR, and SII were log_2_-transformed and are interpreted per doubling. Platelet count was scaled per 50 × 10^9^/L increase.

**Table 3:** Multivariable Firth penalized logistic regression models for in-hospital mortality.

Model	Predictor	aOR (95% CI)	*p*-Value
Core ICU	Age, per 10-year increase	1.17 (0.75–1.83)	0.491
	Liver metastases	6.96 (2.41–21.80)	<0.001
	Respiratory failure	6.78 (2.10–24.10)	0.001
	Other critical illness	6.85 (1.75–32.10)	0.005
Core ICU + NLR	Age, per 10-year increase	1.22 (0.78–1.93)	0.381
	Liver metastases	6.09 (2.06–19.30)	0.001
	Respiratory failure	6.26 (1.93–22.20)	0.002
	Other critical illness	6.20 (1.55–29.60)	0.009
	NLR, per doubling	1.21 (0.87–1.75)	0.264
Core ICU + PLR	Age, per 10-year increase	1.18 (0.75–1.85)	0.471
	Liver metastases	7.61 (2.55–25.10)	<0.001
	Respiratory failure	7.27 (2.21–26.80)	<0.001
	Other critical illness	7.04 (1.79–33.00)	0.005
	PLR, per doubling	0.89 (0.64–1.21)	0.448
Core ICU + SII	Age, per 10-year increase	1.18 (0.75–1.85)	0.469
	Liver metastases	7.40 (2.48–24.20)	<0.001
	Respiratory failure	7.08 (2.17–25.60)	0.001
	Other critical illness	6.95 (1.78–32.20)	0.005
	SII, per doubling	0.95 (0.75–1.16)	0.603
Core ICU + PLT	Age, per 10-year increase	1.33 (0.81–2.21)	0.256
	Liver metastases	9.48 (2.92–35.80)	<0.001
	Respiratory failure	10.40 (2.89–44.30)	<0.001
	Other critical illness	7.54 (1.83–38.10)	0.005
	Platelet count, per 50 × 10^9^/L increase	0.81 (0.67–0.94)	0.006
Core ICU + NLR + PLT	Age, per 10-year increase	1.45 (0.88–2.47)	0.146
	Liver metastases	8.01 (2.42–30.80)	<0.001
	Respiratory failure	10.40 (2.82–46.00)	<0.001
	Other critical illness	6.57 (1.54–34.40)	0.010
	NLR, per doubling	1.39 (0.96–2.07)	0.080
	Platelet count, per 50 × 10^9^/L increase	0.79 (0.66–0.92)	0.002

Legend: aOR, adjusted odds ratio; CI, confidence interval; ICU, intensive care unit; NLR, neutrophil-to-lymphocyte ratio; PLR, platelet-to-lymphocyte ratio; PLT, platelet count; SII, systemic immune-inflammation index. The Core ICU model included age, hepatic metastatic involvement, and primary reason for ICU admission. Primary reason for ICU admission was categorized as neurological deterioration, respiratory failure, or other critical illness, with neurological deterioration as the reference category. Other critical illness comprised hemorrhage, septic shock, other shock, and postoperative admission. NLR, PLR, and SII were log_2_-transformed and are interpreted per doubling. Platelet count was scaled per 50 × 10^9^/L increase.

**Table 4:** APACHE II-adjusted multivariable Firth penalized logistic regression for in-hospital mortality.

Predictor	Adjusted OR (95% CI)	*p*-Value
APACHE II, per 5-point increase	1.05 (0.71–1.56)	0.791
Hepatic metastatic involvement	7.87 (2.41–29.44)	<0.001
Respiratory failure vs. neurological deterioration	11.29 (3.04–50.78)	<0.001
Other critical illness vs. neurological deterioration	7.31 (1.75–36.97)	0.006
Platelet count, per 50 × 10^9^/L increase	0.82 (0.69–0.94)	0.005
NLR, per doubling	1.29 (0.90–1.89)	0.168

Legend: APACHE II, Acute Physiology and Chronic Health Evaluation II; aOR, adjusted odds ratio; CI, confidence interval; ICU, intensive care unit; NLR, neutrophil-to-lymphocyte ratio. APACHE II was scaled per five-point increase, platelet count per 50 × 10^9^/L increase, and NLR was log_2_-transformed.

**Table 5:** Cox proportional hazards analysis of time to in-hospital death—univariable analysis.

Predictor	N	Events	HR (95% CI)	*p*-Value
Age, per 10-year increase	120	96	1.16 (0.96–1.40)	0.124
Liver metastases	120	96	1.85 (1.05–3.27)	0.035
HGB, per 1 g/dL increase	120	96	0.98 (0.92–1.05)	0.638
WBC, per 5 × 10^9^/L increase	120	96	1.13 (1.04–1.23)	0.004
Platelet count, per 50 × 10^9^/L increase	120	96	0.93 (0.87–0.99)	0.026
Creatinine, per unit increase	120	96	1.17 (1.02–1.33)	0.020
Urea, per unit increase	120	96	1.01 (1.00–1.01)	<0.001
NLR, per doubling	120	96	1.20 (1.02–1.41)	0.028
PLR, per doubling	120	96	0.98 (0.84–1.14)	0.783
SII, per doubling	120	96	1.01 (0.92–1.12)	0.816
Respiratory failure vs. neurological deterioration	120	96	1.25 (0.67–2.33)	0.483
Other critical illness vs. neurological deterioration	120	96	2.05 (1.04–4.05)	0.039

Legend: CI, confidence interval; HR, hazard ratio; NLR, neutrophil-to-lymphocyte ratio; PLR, platelet-to-lymphocyte ratio; SII, systemic immune-inflammation index; WBC, white blood cell count.

**Table 6:** Cox proportional hazards analysis of time to in-hospital death—multivariable analysis.

Model	Predictor	aHR (95% CI)	*p*-Value	Global PH *p*-Value
Core ICU	Age, per 10-year increase	1.11 (0.92–1.35)	0.289	0.405
	Liver metastases	2.06 (1.16–3.67)	0.014	
	Respiratory failure	1.30 (0.69–2.45)	0.419	
	Other critical illness	2.14 (1.04–4.38)	0.038	
Core ICU + NLR	Age, per 10-year increase	1.17 (0.96–1.44)	0.121	0.524
	Liver metastases	1.89 (1.06–3.38)	0.031	
	Respiratory failure	1.27 (0.67–2.41)	0.462	
	Other critical illness	2.03 (0.99–4.14)	0.053	
	NLR, per doubling	1.21 (1.02–1.44)	0.026	
Core ICU + PLR	Age, per 10-year increase	1.11 (0.91–1.34)	0.311	0.445
	Liver metastases	2.11 (1.17–3.79)	0.013	
	Respiratory failure	1.33 (0.70–2.53)	0.386	
	Other critical illness	2.16 (1.05–4.44)	0.036	
	PLR, per doubling	0.96 (0.81–1.13)	0.634	
Core ICU + SII	Age, per 10-year increase	1.11 (0.92–1.36)	0.285	0.484
	Liver metastases	2.04 (1.14–3.67)	0.016	
	Respiratory failure	1.29 (0.68–2.45)	0.430	
	Other critical illness	2.13 (1.04–4.37)	0.038	
	SII, per doubling	1.01 (0.91–1.12)	0.879	
Core ICU + PLT	Age, per 10-year increase	1.10 (0.91–1.33)	0.339	0.498
	Liver metastases	2.11 (1.18–3.76)	0.012	
	Respiratory failure	1.41 (0.74–2.68)	0.297	
	Other critical illness	2.09 (1.02–4.30)	0.045	
	Platelet count, per 50 × 10^9^/L increase	0.93 (0.87–1.00)	0.047	
Core ICU + NLR + PLT	Age, per 10-year increase	1.16 (0.96–1.41)	0.134	0.641
	Liver metastases	1.85 (1.03–3.33)	0.039	
	Respiratory failure	1.39 (0.73–2.66)	0.319	
	Other critical illness	1.94 (0.95–3.99)	0.071	
	NLR, per doubling	1.28 (1.08–1.53)	0.005	
	Platelet count, per 50 × 10^9^/L increase	0.91 (0.84–0.98)	0.011	

Legend: aHR, adjusted hazard ratio; CI, confidence interval; ICU, intensive care unit; NLR, neutrophil-to-lymphocyte ratio; PH, proportional hazards; PLR, platelet-to-lymphocyte ratio; PLT, platelet count; SII, systemic immune-inflammation index. The Core ICU model included age, hepatic metastatic involvement, and primary reason for ICU admission. Primary reason for ICU admission was categorized as neurological deterioration, respiratory failure, or other critical illness, with neurological deterioration as the reference category. Other critical illness comprised hemorrhage, septic shock, other shock, and postoperative admission. NLR, PLR, and SII were log_2_-transformed and are interpreted per doubling. Platelet count was scaled per 50 × 10^9^/L increase.

**Table 7:** APACHE II-adjusted multivariable Cox proportional-hazards regression for time to in-hospital death.

Predictor	aHR (95% CI)	*p*-Value
APACHE II, per 5-point increase	1.28 (1.07–1.53)	0.006
Hepatic metastatic involvement	2.06 (1.14–3.74)	0.017
Respiratory failure vs. neurological deterioration	1.61 (0.85–3.05)	0.142
Other critical illness vs. neurological deterioration	2.17 (1.08–4.36)	0.029
Platelet count, per 50 × 10^9^/L increase	0.91 (0.84–0.98)	0.010
NLR, per doubling	1.25 (1.05–1.48)	0.011

Legend: aHR, adjusted hazard ratio; APACHE II, Acute Physiology and Chronic Health Evaluation II; CI, confidence interval; ICU, intensive care unit; NLR, neutrophil-to-lymphocyte ratio.

**Table 8:** APACHE II-adjusted landmark Cox regression analyses at 24 and 48 h following ICU admission.

Predictor	24-h Landmark (n = 113; 90 Deaths), aHR (95% CI)	*p*-Value	48-h Landmark (n = 100; 79 Deaths), aHR (95% CI)	*p*-Value
APACHE II, per 5-point increase	1.20 (0.99–1.44)	0.058	1.16 (0.95–1.42)	0.155
Hepatic metastatic involvement	1.87 (1.03–3.40)	0.040	2.03 (1.07–3.87)	0.031
Respiratory failure vs. neurological deterioration	1.88 (0.94–3.75)	0.072	1.85 (0.90–3.83)	0.096
Other critical illness vs. neurological deterioration	2.52 (1.19–5.33)	0.016	2.43 (1.09–5.44)	0.031
Platelet count, per 50 × 10^9^/L increase	0.88 (0.81–0.96)	0.002	0.90 (0.83–0.98)	0.015
NLR, per doubling	1.31 (1.09–1.57)	0.003	1.24 (1.01–1.50)	0.036

Legend: aHR, adjusted hazard ratio; APACHE II, Acute Physiology and Chronic Health Evaluation II; CI, confidence interval; ICU, intensive care unit; NLR, neutrophil-to-lymphocyte ratio.

**Table 9:** Apparent and bootstrap-corrected performance of Firth logistic models for in-hospital mortality.

Model	Apparent AUROC	Corrected AUROC (95% CI)	Apparent Brier	Corrected Brier (95% CI)	Apparent Slope	Corrected Slope
Core ICU	0.784	0.743 (0.654–0.860)	0.126	0.141 (0.091–0.169)	1.063	0.896
Core ICU + NLR	0.794	0.745 (0.665–0.867)	0.125	0.143 (0.091–0.169)	1.080	0.855
Core ICU + PLR	0.784	0.734 (0.651–0.859)	0.125	0.142 (0.090–0.168)	1.072	0.858
Core ICU + SII	0.786	0.732 (0.650–0.855)	0.126	0.143 (0.091–0.169)	1.070	0.841
Core ICU + PLT	0.838	0.799 (0.731–0.897)	0.116	0.134 (0.086–0.160)	1.095	0.867
Core ICU + NLR + PLT	0.845	0.802 (0.741–0.902)	0.111	0.131 (0.083–0.156)	1.114	0.852

Legend: AUROC, area under the receiver-operating-characteristic curve; CI, confidence interval; ICU, intensive care unit; NLR, neutrophil-to-lymphocyte ratio; PLR, platelet-to-lymphocyte ratio; PLT, platelet count; SII, systemic immune-inflammation index.

**Table 10:** Apparent and bootstrap optimism-corrected predictive performance of APACHE II-adjusted Firth logistic regression models for in-hospital mortality.

Model	Apparent AUROC	Corrected AUROC	Apparent Brier Score	Corrected Brier Score	Apparent Calibration Slope	Corrected Calibration Slope
Severity-adjusted Core	0.787	0.745	0.127	0.141	1.064	0.899
Severity-adjusted Core + PLT	0.831	0.793	0.118	0.134	1.094	0.882
Severity-adjusted Core + PLT + NLR	0.829	0.786	0.113	0.132	1.107	0.858

Legend: APACHE II, Acute Physiology and Chronic Health Evaluation II; AUROC, area under the receiver operating characteristic curve; ICU, intensive care unit; NLR, neutrophil-to-lymphocyte ratio; PLT, platelet count.

**Table 11:** Sensitivity analysis of the multivariable Firth model after exclusion of early in-hospital deaths.

Analysis	Predictor	aOR (95% CI)	*p*-Value
Primary analysis (N = 120; deaths = 96)	Age, per 10-year increase	1.45 (0.88–2.47)	0.146
	Liver metastases	8.01 (2.42–30.75)	<0.001
	Respiratory failure	10.43 (2.82–46.03)	<0.001
	Other critical illness	6.57 (1.54–34.37)	0.010
	NLR, per doubling	1.39 (0.96–2.07)	0.080
	Platelet count, per 50 × 10^9^/L increase	0.79 (0.66–0.92)	0.002
Excluding deaths ≤24 h (N = 114; deaths = 90)	Age, per 10-year increase	1.41 (0.85–2.41)	0.184
	Liver metastases	7.36 (2.23–28.06)	<0.001
	Respiratory failure	11.55 (3.07–51.79)	<0.001
	Other critical illness	6.74 (1.55–36.05)	0.010
	NLR, per doubling	1.36 (0.95–2.02)	0.091
	Platelet count, per 50 × 10^9^/L increase	0.79 (0.66–0.92)	0.002
Excluding deaths ≤48 h (N = 103; deaths = 79)	Age, per 10-year increase	1.38 (0.84–2.34)	0.201
	Liver metastases	7.12 (2.14–27.18)	0.001
	Respiratory failure	10.63 (2.84–47.20)	<0.001
	Other critical illness	6.17 (1.39–33.52)	0.016
	NLR, per doubling	1.31 (0.89–1.98)	0.165
	Platelet count, per 50 × 10^9^/L increase	0.81 (0.68–0.94)	0.006

Legend: aOR, adjusted odds ratio; CI, confidence interval; ICU, intensive care unit; NLR, neutrophil-to-lymphocyte ratio.

**Table 12:** Sensitivity analysis of APACHE II-adjusted Firth logistic regression following exclusion of early in-hospital deaths.

Predictor	Full Cohort (n = 120; 96 Deaths)	Excluding Deaths ≤24 h (n = 114; 90 Deaths)	Excluding Deaths ≤48 h (n = 103; 79 Deaths)
APACHE II, per 5-point increase	1.05 (0.71–1.56); *p* = 0.791	0.95 (0.62–1.46); *p* = 0.832	0.94 (0.62–1.43); *p* = 0.786
Hepatic metastatic involvement	7.87 (2.41–29.44); *p* < 0.001	7.01 (2.14–25.99); *p* = 0.0012	6.74 (2.06–24.86); *p* = 0.0015
Platelet count, per 50 × 10^9^/L increase	0.82 (0.69–0.94); *p* = 0.005	0.81 (0.68–0.94); *p* = 0.004	0.83 (0.70–0.96); *p* = 0.011
NLR, per doubling	1.29 (0.90–1.89); *p* = 0.168	1.30 (0.91–1.91); *p* = 0.146	1.26 (0.86–1.89); *p* = 0.232

Legend: APACHE II, Acute Physiology and Chronic Health Evaluation II; NLR, neutrophil-to-lymphocyte ratio.

**Table 13:** In-hospital biological evolution according to survival status.

Characteristic	Overall	Survivors	Non-Survivors	*p*-Value	FDR-Adjusted *p*	N Available
Creatinine						
Baseline	1.42 [0.95–1.78]	1.39 [1.02–1.69]	1.42 [0.94–1.84]	0.798	**<0.001**	120
Final	1.59 [1.08–2.83]	1.02 [0.91–1.46]	1.97 [1.24–3.19]	**<0.001**		120
Change	0.19 [−0.11–0.80]	−0.14 [−0.41–0.00]	0.42 [−0.02–1.01]	**<0.001**		120
Urea						
Baseline	70.50 [40.00–104.75]	68.00 [35.50–77.50]	72.50 [41.50–110.25]	0.180	**0.005**	120
Final	101.00 [58.00–150.50]	61.00 [23.00–75.75]	110.00 [61.75–160.25]	**<0.001**		120
Change	19.00 [−5.25–59.25]	0.00 [−16.00–12.75]	28.00 [−1.00–66.25]	**0.002**		120
INR						
Baseline	1.38 [1.18–1.63]	1.31 [1.16–1.46]	1.41 [1.18–1.65]	0.210	**0.041**	116
Final	1.60 [1.33–1.93]	1.32 [1.21–1.50]	1.65 [1.39–2.04]	**<0.001**		115
Change	0.14 [−0.07–0.44]	−0.07 [−0.12–0.27]	0.15 [−0.02–0.52]	**0.041**		115
AST						
Baseline	37.00 [22.00–80.00]	27.50 [19.75–33.50]	45.00 [24.00–93.00]	**0.016**	**0.016**	120
Final	46.00 [24.00–154.50]	21.50 [17.75–30.25]	60.50 [29.00–223.25]	**<0.001**		120
Change	2.00 [−13.25–52.50]	−1.50 [−15.25–0.50]	6.50 [−12.25–91.00]	**0.012**		120

Legend: AST, aspartate aminotransferase; FDR, false discovery rate; INR, international normalized ratio; IQR, interquartile range.

**Table 14:** Spearman correlations among inflammation-based indices.

Inflammatory Index	NLR	PLR	SII
NLR	1.000	0.517	0.804
PLR	0.517	1.000	0.686
SII	0.804	0.686	1.000

Legend: LMR, lymphocyte-to-monocyte ratio; NLR, neutrophil-to-lymphocyte ratio; PLR, platelet-to-lymphocyte ratio; SII, systemic immune-inflammation index.

**Table 15:** Cumulative incidence of in-hospital death and discharge alive.

Time After ICU Admission	In-Hospital Death, % (95% CI)	Discharge Alive, % (95% CI)
Day 1	5.0 (1.1–8.9)	0.8 (0.0–2.5)
Day 3	22.5 (15.0–30.0)	3.3 (0.1–6.6)
Day 7	48.3 (39.3–57.3)	7.5 (2.8–12.2)
Day 14	66.7 (58.1–75.2)	13.3 (7.2–19.5)
Day 21	75.8 (68.0–83.6)	17.5 (10.6–24.4)
Day 28	77.5 (69.9–85.1)	18.3 (11.3–25.4)

Legend: CI, confidence interval; ICU, intensive care unit.

**Table 16:** Fine–Gray competing-risk regression for in-hospital death.

Predictor	sHR (95% CI)	*p*-Value
Age, per 10-year increase	1.19 (0.99–1.43)	0.065
Liver metastases	2.33 (1.37–3.97)	0.002
Respiratory failure	2.02 (0.98–4.18)	0.058
Other critical illness	2.32 (1.00–5.39)	0.050
NLR, per doubling	1.25 (1.07–1.45)	0.004
Platelet count, per 50 × 10^9^/L increase	0.90 (0.84–0.97)	0.003

Legend: CI, confidence interval; ICU, intensive care unit; NLR, neutrophil-to-lymphocyte ratio; sHR, subdistribution hazard ratio.

**Table 17:** APACHE II-adjusted Fine–Gray competing-risk regression for in-hospital death.

Predictor	sHR (95% CI)	*p*-Value
APACHE II, per 5-point increase	1.32 (1.10–1.58)	0.003
Hepatic metastatic involvement	2.70 (1.46–4.98)	0.001
Respiratory failure vs. neurological deterioration	2.37 (1.09–5.12)	0.029
Other critical illness vs. neurological deterioration	2.67 (1.13–6.31)	0.025
Platelet count, per 50 × 10^9^/L increase	0.90 (0.84–0.97)	0.008
NLR, per doubling	1.20 (1.03–1.40)	0.018

Legend: APACHE II, Acute Physiology and Chronic Health Evaluation II; CI, confidence interval; ICU, intensive care unit; NLR, neutrophil-to-lymphocyte ratio; sHR, subdistribution hazard ratio. APACHE II was scaled per five-point increase, platelet count per 50 × 10^9^/L increase, and NLR was log_2_-transformed.

**Table 18:** APACHE II-adjusted Fine–Gray competing-risk landmark analyses at 24 and 48 h following ICU admission.

Predictor	24-h Landmark (n = 113), sHR (95% CI)	*p*-Value	48-h Landmark (n = 100), sHR (95% CI)	*p*-Value
APACHE II, per 5-point increase	1.23 (1.02–1.48)	0.033	1.21 (0.97–1.50)	0.090
Hepatic metastatic involvement	2.41 (1.33–4.37)	0.004	2.31 (1.24–4.29)	0.008
Respiratory failure vs. neurological deterioration	2.77 (1.20–6.40)	0.017	2.76 (1.13–6.76)	0.026
Other critical illness vs. neurological deterioration	2.98 (1.18–7.56)	0.021	3.08 (1.14–8.30)	0.026
Platelet count, per 50 × 10^9^/L increase	0.88 (0.82–0.95)	0.001	0.90 (0.83–0.97)	0.008
NLR, per doubling	1.24 (1.05–1.47)	0.013	1.184 (1.004–1.396)	0.044

Legend: APACHE II, Acute Physiology and Chronic Health Evaluation II; CI, confidence interval; ICU, intensive care unit; NLR, neutrophil-to-lymphocyte ratio; sHR, subdistribution hazard ratio.

## Data Availability

The datasets are available from the correspondent authors upon a reasonable request due to local policies.

## Supplementary Materials

The following supporting information can be downloaded at https://www.mdpi.com/article/10.3390/jcm15218669/s1, Table S1: Pairwise differences in optimism-corrected discrimination and overall prediction error; Table S2: Decision-curve analysis: incremental optimism-corrected net benefit of hematological predictors in APACHE II-adjusted models; Figure S1: Calibration plots of the original clinical Firth logistic regression models for in-hospital mortality; Figure S2: Calibration plots of APACHE II-adjusted Firth logistic regression models for in-hospital mortality; Figure S3: Exploratory decision-curve analysis of the original clinical prediction models for in-hospital mortality; Figure S4: Exploratory decision-curve analysis of APACHE II-adjusted prediction models for in-hospital mortality.
