# Supplementary material for: Clinical, Oncological, and Hematological Determinants of In-Hospital Mortality in Critically Ill Patients with Advanced Neoplasia: A Retrospective Cohort Study
Source: J Clin Med. 2026 Nov 6;15(21):8669. doi: 10.3390/jcm15218669 (PMC13763766; doi:10.3390/jcm15218669)
Supplement: Supplementary file 1 [file jcm-15-08669-s001.zip › jcm-4614171-supplementary/Figure S2.pdf]

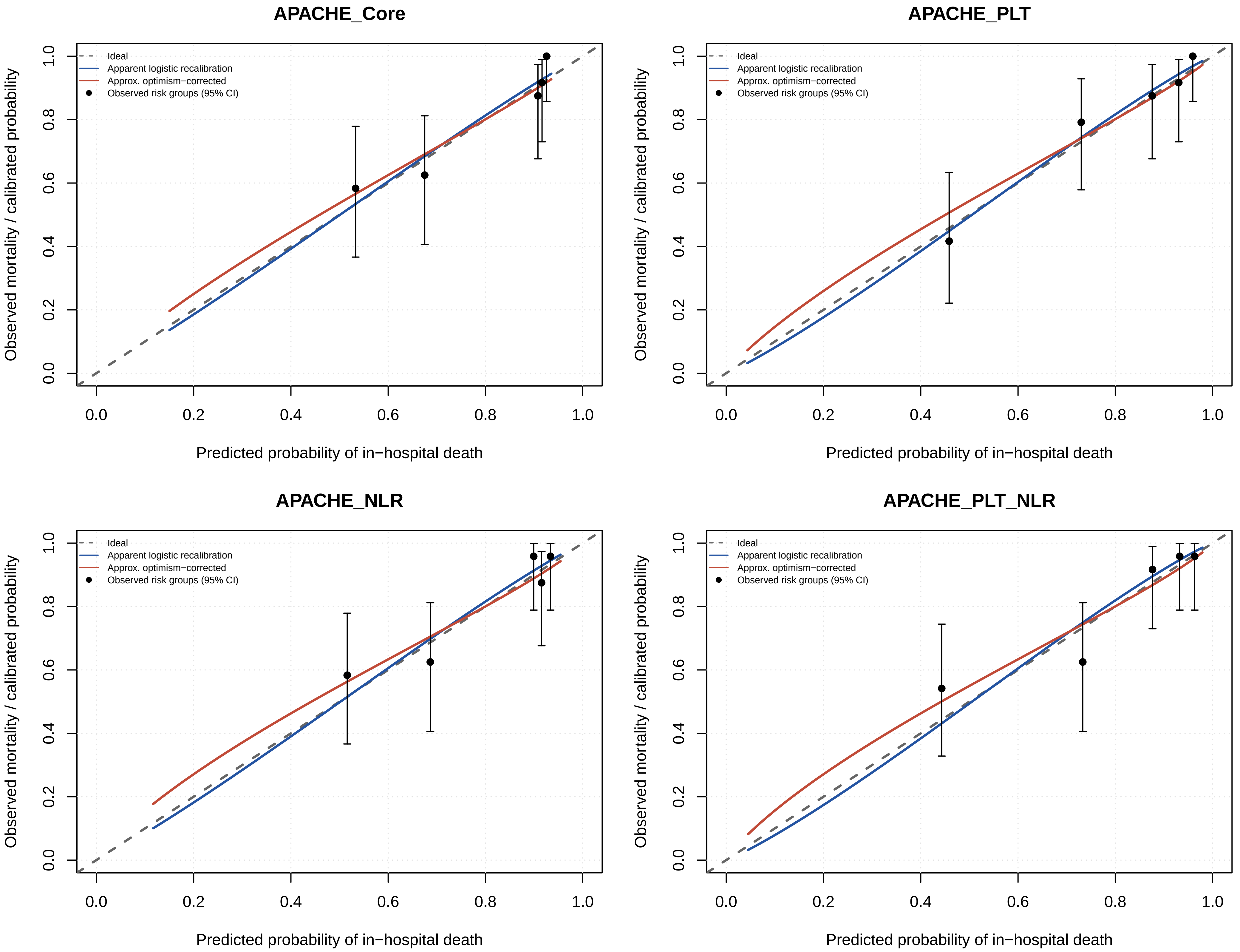
APACHE_Core
APACHE_PLT
APACHE_NLR
APACHE_PLT_NLR
Ideal
Apparent logistic recalibration
Approx. optimism−corrected
Observed risk groups (95% CI)
Observed mortality / calibrated probability
Predicted probability of in−hospital death
0.0
0.2
0.4
0.6
0.8
1.0
