# Supplementary material for: Clinical, Oncological, and Hematological Determinants of In-Hospital Mortality in Critically Ill Patients with Advanced Neoplasia: A Retrospective Cohort Study
Source: J Clin Med. 2026 Nov 6;15(21):8669. doi: 10.3390/jcm15218669 (PMC13763766; doi:10.3390/jcm15218669)
Supplement: Supplementary file 1 [file jcm-15-08669-s001.zip › jcm-4614171-supplementary/Tables S1 and S2.pdf]

Supplementary Table S1. Pairwise differences in optimism-corrected discrimination and overall prediction error

Comparison (extended – reference)	ΔAUROC (95% CI)	ΔBrier score (95% CI)
Clinical Core + NLR vs. Core	+0.002 (−0.018–0.065)	+0.002 (−0.012–0.004)
Clinical Core + PLR vs. Core	−0.009 (−0.027–0.036)	+0.001 (−0.011–0.003)
Clinical Core + SII vs. Core	−0.010 (−0.028–0.036)	+0.002 (−0.010–0.003)
Clinical Core + PLT vs. Core	+0.056 (0.002–0.146)	−0.007 (−0.032–0.003)
Clinical Core + PLT + NLR vs. Core	+0.059 (0.004–0.154)	−0.010 (−0.039–0.003)
Clinical Core + PLT + NLR vs. Core + PLT	+0.004 (−0.012–0.044)	−0.003 (−0.021–0.003)
APACHE Core + NLR vs. Core	−0.021 (−0.037–0.048)	+0.001 (−0.012–0.003)
APACHE Core + PLT vs. Core	+0.048 (−0.0003–0.128)	−0.008 (−0.028–0.003)
APACHE Core + PLT + NLR vs. Core	+0.041 (−0.007–0.128)	−0.009 (−0.036–0.003)
APACHE Core + PLT + NLR vs. Core + PLT	−0.007 (−0.019–0.031)	−0.002 (−0.018–0.003)

Legend: APACHE II, Acute Physiology and Chronic Health Evaluation II; AUROC, area under the receiver operating characteristic curve; CI, confidence interval; NLR, neutrophil-to-lymphocyte ratio; PLR, platelet-to-lymphocyte ratio; PLT, platelet count; SII, systemic immune-inflammation index. The Clinical Core model included age, hepatic metastatic involvement, and primary reason for ICU admission. The APACHE II-adjusted Core model replaced age with APACHE II. All models were estimated using Firth penalized logistic regression and internally validated using 1000 paired bootstrap resamples, with identical resampling indices applied across candidate models. Approximate 95% confidence intervals for optimism-corrected AUROC and Brier scores, and their pairwise differences, were calculated using a location-shifted percentile bootstrap approach. Calibration slopes are reported as optimism-corrected point estimates.

Supplementary Table S2. Decision-curve analysis: incremental optimism-corrected net benefit of hematological predictors in APACHE II-adjusted models

Comparison (extended – reference)	Threshold probability	Δ Net benefit (95% CI)
APACHE Core + PLT vs. Core	0.60	+0.087 (−0.034–0.120)
	0.70	+0.013 (−0.051–0.146)
	0.80	+0.015 (−0.093–0.182)
	0.85	+0.022 (−0.111–0.206)
APACHE Core + NLR vs. Core	0.60	+0.043 (−0.049–0.072)
	0.70	−0.020 (−0.058–0.089)
	0.80	−0.018 (−0.084–0.091)
	0.85	−0.028 (−0.130–0.122)
APACHE Core + PLT + NLR vs. Core	0.60	+0.064 (−0.040–0.131)
	0.70	+0.060 (−0.046–0.162)
	0.80	+0.028 (−0.097–0.186)
	0.85	+0.011 (−0.114–0.214)
APACHE Core + PLT + NLR vs. Core + PLT	0.60	−0.023 (−0.052–0.069)
	0.70	+0.047 (−0.056–0.097)
	0.80	+0.013 (−0.087–0.121)
	0.85	−0.011 (−0.106–0.136)

Legend: APACHE II, Acute Physiology and Chronic Health Evaluation II; CI, confidence interval; NLR, neutrophil-to-lymphocyte ratio; PLT, platelet count. Estimates were corrected for optimism using paired bootstrap resampling (1000 replicates), with approximate 95% confidence intervals. Decision-curve analysis was explored over threshold probabilities from 0.50 to 0.85.
